# [^18^F]AZD2461, an Insight on Difference in PARP Binding Profiles for DNA Damage Response PET Imaging

**DOI:** 10.1007/s11307-020-01497-6

**Published:** 2020-04-27

**Authors:** Florian Guibbal, Samantha L. Hopkins, Anna Pacelli, Patrick G. Isenegger, Michael Mosley, Julia Baguña Torres, Gemma M. Dias, Damien Mahaut, Rebekka Hueting, Véronique Gouverneur, Bart Cornelissen

**Affiliations:** 1 Department of Oncology, CRUK/MRC Oxford Institute for Radiation Oncology, University of Oxford, Old Road Campus Research Building , Off Roosevelt Drive, Oxford, OX3 7LJ UK; 2 Department of Chemistry, Chemistry Research Laboratory, University of Oxford, 12 Mansfield Road, Oxford, OX1 3TA UK

**Keywords:** PET, AZD2461, PARP, Cancer, Molecular imaging

## Abstract

**Background:**

Poly (ADP-ribose) polymerase (PARP) inhibitors are extensively studied and used as anti-cancer drugs, as single agents or in combination with other therapies. Most radiotracers developed to date have been chosen on the basis of strong PARP1–3 affinity. Herein, we propose to study AZD2461, a PARP inhibitor with lower affinity towards PARP3, and to investigate its potential for PARP targeting *in vivo*.

**Methods:**

Using the Cu-mediated ^18^F-fluorodeboronation of a carefully designed radiolabelling precursor, we accessed the ^18^F-labelled isotopologue of the PARP inhibitor AZD2461. Cell uptake of [^18^F]AZD2461 *in vitro* was assessed in a range of pancreatic cell lines (PSN-1, PANC-1, CFPAC-1 and AsPC-1) to assess PARP expression and *in vivo* in xenograft-bearing mice. Blocking experiments were performed with both olaparib and AZD2461.

**Results:**

[^18^F]AZD2461 was efficiently radiolabelled *via* both manual and automated procedures (9 % ± 3 % and 3 % ± 1 % activity yields non-decay corrected). [^18^F]AZD2461 was taken up *in vivo* in PARP1-expressing tumours, and the highest uptake was observed for PSN-1 cells (7.34 ± 1.16 %ID/g). *In vitro* blocking experiments showed a lesser ability of olaparib to reduce [^18^F]AZD2461 binding, indicating a difference in selectivity between olaparib and AZD2461.

**Conclusion:**

Taken together, we show the importance of screening the PARP selectivity profile of radiolabelled PARP inhibitors for use as PET imaging agents.

**Electronic supplementary material:**

The online version of this article (10.1007/s11307-020-01497-6) contains supplementary material, which is available to authorized users.

## Background

Genomic instability in cancerous tissues is increased as a result of oncogenic and replicative stress, exogenous genotoxic insults and tumour-specific DNA repair defects [1]. Manipulating genomic instability can therefore provide therapeutic opportunities, and inhibitors of DNA damage repair enzymes have been extensively explored as anti-cancer drugs [2]. Among these, poly (ADP-ribose) polymerase (PARP) inhibitors have advanced furthest in the clinic. Selective PARP inhibitors act on PARP1, PARP2 and PARP3, all members of the 17-member PARP enzyme family, also referred to as ART diphtheria toxin-like (ARTD) enzymes, which also includes Tankyrase-1 and -2. PARP1–3 bind to single-strand damaged DNA and play an important role in its repair *via* the base excision repair (BER) pathway. PARP inhibitors, such as olaparib (Lynparza), niraparib (Zejula) and rucaparib (Rubraca) reduce catalytic activity and interfere with the ability of the enzyme to dislodge from DNA [3, 4]. They have been extensively studied as single agents and radiation sensitizers and are especially effective in tumours with defects in homologous recombination (HR) DNA damage repair (DDR), such as mutations in BReast CAncer gene (BRCA) [5, 6]. The potential of PARP inhibitors was first shown in breast cancer associated (BRCA)-deficient cancer such as breast or ovarian cancer [7]. Olaparib (ku-0059436, AZ2281, Lynparza®) was the first FDA approved oral potent PARP inhibitor that inhibits PARP isoforms 1 and 2 and, to a lesser extent, PARP3 [8]. To date, more than 100 clinical trials are currently ongoing utilizing olaparib therapy as a single agent or as a combination with chemo-, immuno- and radionuclide therapies. In these clinical trials, interruption of the treatment was mainly due to tumour progression, thought to be closely related to emerging drug resistance [9]. Resistance to PARP therapies is common, but not clearly understood. Reports have shown that between 30 and 70 % of treated patients having DDR machinery (HR) defects are not responsive to PARP inhibitor therapy [10]. One of the mechanisms thought to be involved in PARP inhibitor resistance is overexpression of Abcb1a and Abcb1b genes encoding efflux membrane transporter *P*-glycoprotein (*P*-gp). Studies have shown overexpression of these genes in BRCA1- and BRCA2-deficient mouse mammary tumours which is thought to be associated with resistance to olaparib in this model [11, 12].

Positron emission tomography (PET) is a powerful diagnostic tool providing molecular and functional information, based on compounds radiolabelled with positron-emitting radionuclides, such as ^11^C or ^18^F. PET imaging, an extremely sensitive, non-invasive technique that is able to provide real-time information, has proven to be a very useful tool for measuring PARP expression and the pharmacokinetics and dynamics of radiolabelled PARP inhibitors *in vivo* [13, 14]. Fluorine-18 is the most widely used PET radionuclide due to its appropriate half-life (*t*_1/2_ = 109.8 min), good spatial resolution and—most importantly—due to the stability of the C–F bond [15, 16]. A number of groups have demonstrated the potential of ^18^F-labelled PARP inhibitors in preclinical studies [17–20], reviewed in [13] and [14]. [^18^F]PARPi is a ^18^F-labelled PARP structurally related to olaparib [21]. To date, together with the rucaparib-related tracer [^18^F]FTT, it is one of the few labelled PARP inhibitors whose structure remains closely related to the native inhibitor that has been investigated in clinical trials [20, 22]. While other labelled compounds have been investigated, such as [^18^F]BO or [^18^F]PARPi-FL, few radiotracers have been studied for their PARP PET imaging potential with a view to assess a different PARP isoform interaction profile [23–25]. A radiolabelled PARP inhibitor that possesses a different PARP inhibitory profile may allow for a better understanding of PARP expression *in vivo*.

**Fig. 1.**
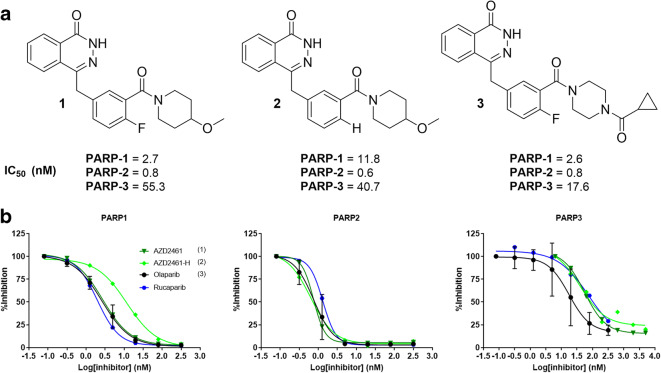
**a** Structures and **b** PARP1, 2 and 3 inhibitory profiles of AZD2461 1, proto-deborylated side-product AZD2461-H 2 and olaparib 3. The PARP inhibitor Rucaparib was added as for comparison.

The PARP inhibitor AZD2461, a structural variant of olaparib, was developed in part to address the limitations of PARP resistance mechanism and bone marrow toxicity (Fig. 1) [26]. AZD2461 has been shown to have a different PARP enzyme catalytic inhibitory profile than olaparib, in particular a lower affinity for PARP3, as well as being a poor *P*-gp substrate. A radiolabelled isotopologue of AZD2461 offers the ability to investigate the role of PARP3 in DDR and more specifically PARP imaging, as well as a wider range of possibilities when developing new compounds that possess different PARP specificity. In 2019, Mach and co-workers described a radiofluorinated compound that was structurally related to AZD2461 obtained through classic ^18^F-radiochemistry [27]. Although bearing similar backbone structure, deviation from parent compound and subsequent lack of knowledge regarding its interaction with PARP1–3 limits the utility of this compound for PET imaging.

Here, we describe the radiofluorination of [^18^F]AZD2461 *via* Cu-mediated ^18^F-fluorodeboronation to result in an isotopologue. We report on the ability of this radiotracer for PARP imaging in PARP-expressing cell model and *in vivo* in a xenograft tumour mouse model of pancreatic cancer. Our study aimed at providing an insight into the importance of the PARP selectivity profile for PARP imaging using PET and revealed a marked difference in specificity between AZD2461 and olaparib.

## Methods

Full materials and methods are presented in the supplemental information accompanying this manuscript.

### Synthesis

**Fig. 2.**
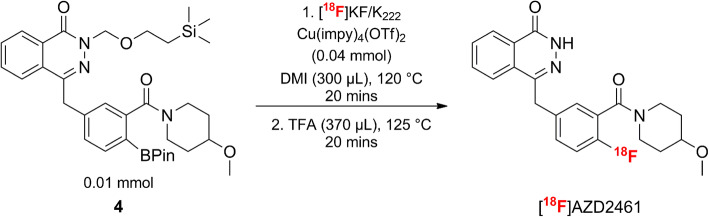
Synthesis of [^18^F]AZD2461 from SEM-protected boronate precursor 4 *via* Cu-mediated radiofluorination.

[^18^F]AZD2461 was radiolabelled *via* the Cu-mediated ^18^F-fluorodeboronation of the corresponding boronic ester precursor (Fig. 2), using previously published methodologies, with minor modifications [17]. The corresponding proto-deborylated side-product and the cold, non-radiolabelled reference, AZD2461, were synthesised *via* a similar pathway (Figs. 1 and 2). [^18^F]AZD2461 was also obtained *via* a fully automated method, using a previously described procedure, and then applied for [^18^F]olaparib [28]. See Supplemental Information for a full description of both radiosyntheses.

### *In Vitro* Evaluation

Pancreatic ductal adenocarcinoma (PDAC) cell lines PSN-1, PANC-1, CFPAC-1 and AsPC-1 were originally purchased from ATCC. Cells were cultured in Dulbecco’s Modified Eagle Medium (DMEM), supplemented with 10 % foetal bovine serum (FBS), 2 mM *L*-glutamine, 100 units/ml penicillin and 0.1 mg/ml streptomycin, in a 37 °C environment containing 5 % CO_2_. Cells were authenticated by the provider, and their identity was corroborated by STR profiling. The cumulative length of culture was less than 3 months following retrieval from liquid nitrogen storage. Cells were tested regularly to confirm the absence of mycoplasma contamination.

The ability of AZD2461, a protonated version, and olaparib (compounds 1, 2 and 3) to inhibit PARP1, 2 and 3 was assessed using a cell-free chemiluminescent assay kit, evaluating the inhibition of enzyme-mediated PARylation of histones (cat. No 80551, BPS Bioscience), as per the manufacturer’s instructions. Briefly, 96 well-assay plates were coated with histone proteins, washed and blocked at room temperature for 1 h. Next, the ribosylation reaction mixture containing the biotinylated enzyme substrate was added to the plate, followed by serial dilutions of the appropriate inhibitor. The reaction was initiated with the addition of the specific PARP enzyme (PARP1–3). Finally, the plate was treated with streptavidin-HRP followed by the HRP substrate to produce chemiluminescence that was measured in a plate reader (Tecan Infinite M200 Pro). All statistical analyses and non-linear regressions were performed using GraphPad Prism v8 (GraphPad Software).

Relative PARP1 levels were determined using Western blot. In brief, total cell lysates from 1 × 10^7^ cells (AsPC-1, CFPAC-1, PANC-1 and PSN-1) were prepared using standard RIPA lysis buffer (50 mM Tris, pH 8, 1 % NP40, 0.5 % sodium deoxycholate, 0.1 % sodium dodecyl sulphate, 150 mM sodium chloride plus cOmplete™ protease inhibitor cocktail [Sigma-Aldrich]). The cell lysates were isolated by centrifugation after lysis through a 21G hypodermic syringe. Fifteen micrograms of total protein were run on a 4–12 % Bis-Tris MOPS gel (Novex) and transferred to a PVDF membrane (Invitrogen iBlot-2). The blots were blocked in 5 % BSA at room temperature for 1 h and exposed to a 1:500 dilution of anti-PARP1 antibody (Atlas HPA045168) at 4 °C overnight, followed by a 1:3000 dilution of the secondary goat anti-rabbit-HRP antibody (R&D Systems HAF008) at room temperature for 1 h. Sample loading controls were performed using an anti-beta actin antibody (Abcam ab8227). The blots were developed using the SuperSignal West Pico PLUS Chemiluminescent Substrate kit (#34580, Thermo Scientific) and exposed to a Li-Cor 3600 Blot Scanner.

To evaluate PARP-mediated cell uptake, aliquots of 5 × 10^5^ cells per well were seeded in 24 well plates and allowed to adhere overnight. Cells were exposed to [^18^F]AZD2461 (50 kBq/well) for 1 h at 37 °C. After washing, cells were lysed (0.1 M NaOH, 10 min), and cell-associated ^18^F was assessed using an automated gamma counter (PerkinElmer Wizard^2^ 2480). To determine the specificity of cell uptake, an excess of cold, unlabelled olaparib or AZD2461 (100 μM) was co-incubated with the radiolabelled compound.

### *In Vivo* Evaluation

All animal procedures were performed in accordance with the UK Animals (Scientific Procedures) Act 1986 and with local ethical committee approval. Tumour xenografts were generated by subcutaneous injection of PSN-1, PANC-1, CFPAC-1 or AsPC-1 cell suspensions in the hind flank of Balb/c *nu/nu* mice. A single tumour was implanted per animal. Tumour xenografts were allowed to grow to a size of 200–400 mm^3^ before subsequent procedures.

PET/CT images were acquired 1 h after an intravenous bolus administration of [^18^F]AZD2461 (5–6 MBq) using a VECTor^4^CT scanner (MILabs, Utrecht, the Netherlands). Animals were anaesthetised by 4 % isoflurane gas (0.5 L/min O_2_) and maintained at 2 % and 37 °C throughout the imaging session. The temperature of the animals was maintained at 37 °C, using a custom-built mouse cradle. Image acquisition was performed in 10 min using a 1.8-mm pinhole collimator. Whole-body CT images were acquired at a tube setting of 55 kVp, 0.19 mA and 20 ms per view, for anatomical reference and attenuation correction. Reconstruction of both CT and PET images was performed with the MILabs reconstruction analysis using a γ-ray energy window of 467–571 keV (background weight 2.5 %), 0.6 mm^3^ voxel size, 128 subsets and 5 iterations using the manufacturer’s SROSEM reconstruction type. PET images were each registered to CT and then attenuation corrected. Images were calibrated by imaging a phantom containing a fluorine-18 standard solution and analysed using PMod software package (Version 3.807, PMOD Technologies). Biodistribution studies, where selected tissues were harvested, were performed immediately after imaging. Some animals were also intravenously administered an excess of cold, unlabelled olaparib or AZD2461 (0.02 mg, 100 μL) 30 min before [^18^F]AZD2461 injection, to evaluate the specificity of tumour uptake. The amount of [^18^F]AZD2461 uptake in selected tissue was determined and reported as a percentage of the injected dose per gram of tissue (%ID/g). Three mice were utilised per group.

### Statistical Analysis

All statistical analyses and nonlinear regression were performed using GraphPad Prism v8 (GraphPad Software, San Diego, CA, USA). Data were tested for normality and analysed by 2-way or 1-way analysis of variance (ANOVA), with Tukey’s post-tests, as appropriate, to calculate the significance of differences between groups. All data were obtained at least in triplicate and results reported as mean ± standard deviation, unless indicated otherwise.

## Results

### [^18^F]AZD261 Labelling *via* Copper-Mediated ^18^F-Fluorodeboronation of Aryl Boronate Ester

[^18^F]AZD2461 was prepared *via* copper-mediated ^18^F-fluorodeboronation of a protected boronic pinacol ester precursor (Fig. 2, Supplemental Fig. 1) in an activity yield of 9 % ± 3 % (non-decay corrected, *n* = 5, total synthesis time = 135 min from dried [^18^F]fluoride to reformulated compound) with molar activities up to 17 GBq/μmol and radiochemical purity > 99 %. As shown in our previously reported studies [17, 29], free nitrogens can be detrimental to Cu-mediated radiofluorination. Since the boronic ester precursor used here bears a phtalazinone, appropriate *N*-[2-(trimethylsilyl)ethoxymethyl] (SEM) protection was performed as predicted by previous screening experiments [17]. Conditions previously optimised for radiolabelling of [^18^F]olaparib were used here for ^18^F-fluorodeboronation of [^18^F]AZD2461. The copper catalyst for ^18^F-fluorodeboronation chosen for this study was Cu(OTf)_2_(impy)_4_ (impy, imidazo[1,2-*b*]pyridazine). 1,3-Dimethyl-2-imidazolidinone (DMI) was demonstrated to be compatible and suitable for ^18^F-radiofluorination. More importantly, an undesired side-product—due to protodeboronation of the precursor—could be separated from the desired radiotracer (Supplemental Fig. 4) and allowed isolation of the pure [^18^F]AZD2461 (Supplemental Figs. 5, 6). The final tracer was reformulated in 10 % dimethyl sulfoxide (DMSO) in phosphate-buffered saline (PBS), suitable for subsequent *in vitro* and *in vivo* experiments (600 MBq/ml). A fully automated procedure, using an Eckert & Ziegler Modular-Lab platform, allowed isolation of [^18^F]AZD2461 in an activity yield of 3 % ± 1 % (non-decay corrected, *n* = 4, total synthesis time = 120 min from dried [^18^F]fluoride to reformulated compound) with molar activities up to 237 GBq/μmol and radiochemical purity > 99 %. [^18^F]olaparib was synthesised, for comparison, as described in our previous study [17].

### [^18^F]AZD2461 Is Taken Up Specifically in PARP1 Expressing Xenografts *In Vivo*

**Fig. 3.**
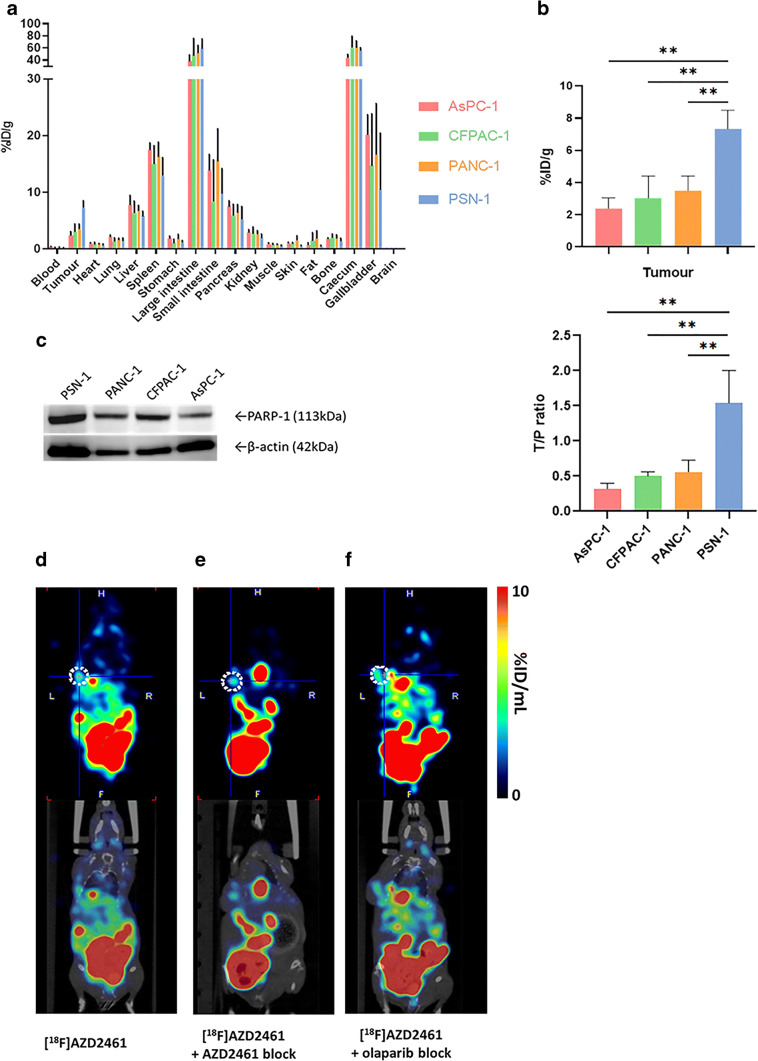
**a** Biodistribution in mice bearing PSN-1, CFPAC-1, AsPC-1 or PANC-1 tumour xenografts, at 1 h *post*-injection of [^18^F]AZD2461. **b** Tumour uptake and tumour/pancreas ratios in PSN-1, CFPAC-1, AsPC-1 and PANC-1 xenografts 1 h *post*-injection **c** Western blot probing for PARP-1 in PSN-1, PANC-1, CFPAC-1 and AsPC-1 cell lines. (**P < 0.01).

**Fig. 4.**
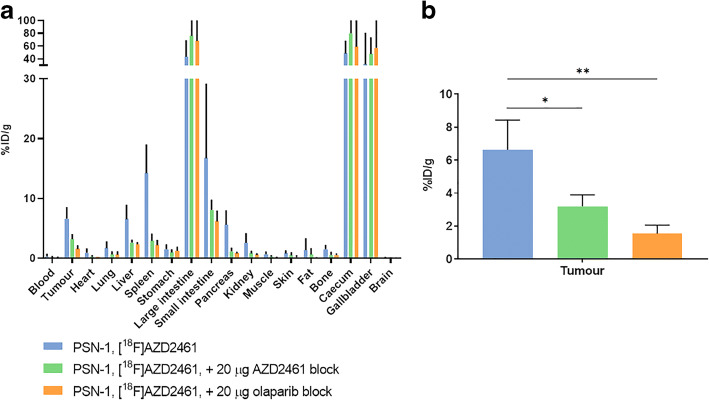
Blocking experiments performed in PSN-1 xenograft-bearing mice. Cold, unlabelled AZD2461 or olaparib (20 μg) was administered intravenously, 30 min prior to [^18^F]AZD2461. **a** Biodistribution, **b** tumour uptake.

To investigate PARP targeting in tumour tissue, mice bearing subcutaneous xenografts were injected intravenously with [^18^F]AZD2461. The biodistribution and excretion pathway was similar to that observed in our previously reported study with [^18^F]olaparib, with [^18^F]AZD2461 showing lower uptake in liver, while splenic uptake values are higher compared with [^18^F]olaparib, at the time point investigated (Fig. 3). Uptake in organs expressing PARP1, 2 and 3 such as the spleen, bone and the pancreas was observed, as well as hepatobiliary excretion. The biodistribution profile of [^18^F]AZD2461 observed in all normal tissues was similar for mice bearing AsPC-1, CFPAC-1 or PANC-1 xenografts (*P* > 0.05). Uptake of [^18^F]AZD2461 in PARP1-expressing xenografts was 2.39 ± 0.65, 3.04 ± 1.37 and 3.50 ± 0.91 %ID/g for AsPC-1, CFPAC-1 and PANC-1, respectively, 1 h after intravenous bolus injection. The highest uptake in tumour was observed in PSN-1 xenografts (7.34 ± 1.16%ID/g) and was significantly higher than that in AsPC-1, CFPAC-1 and PANC-1 xenografts (*P* = 0.0019, 0.0047 and 0.0092, respectively), correlating with higher PARP1 expression levels obtained by Western blot. To investigate the specificity of tumour uptake, injection of an excess of cold, unlabelled olaparib and AZD2461 were performed in mice bearing PSN-1 xenografts. While an excess of olaparib was able to efficiently block tumour uptake of [^18^F]AZD2461 (1.55 ± 0.51 %ID/g, *P* = 0.0022), the same amount of cold AZD2461 only blocked [^18^F]AZD2461 accumulation in tumours to 3.21 ± 0.70 %ID/g (*P* = 0.0204, Fig. 4).

### [^18^F]AZD2461 Is Taken up by PARP-Expressing PSN-1 Cells, with Off-Target Specific Binding

**Fig. 5.**
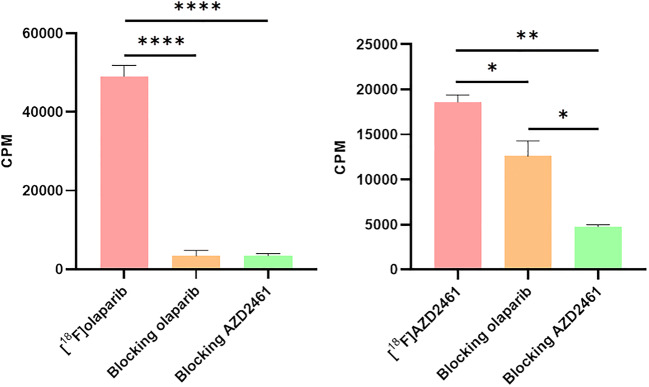
*In vitro* uptake of [^18^F]AZD2461 and [^18^F]olaparib in PSN-1 cells, 30 min after addition of [^18^F]AZD2461. An excess of cold, unlabelled olaparib or AZD2461 was added (100 μM). (* *P* < 0.05, ** *P* < 0.01, **** *P* < 0.0001).

AZD2461 inhibited PARP1–3 enzymatic activity in line with previously reported values (Fig. 1). Proto-deborylated side-product 2 showed an ability to inhibit all isoforms tested. To assess PARP targeting, PARP-expressing pancreatic tumour cells (PSN-1) were exposed to [^18^F]AZD2461 or [^18^F]olaparib for 30 min. Both radiotracers were efficiently taken up in PSN-1 cells (Fig. 5). Uptake of [^18^F]olaparib was similar to that reported in our previously reported study [17]. Blocking experiments showed that addition of an excess of non-radioactive olaparib or AZD2461 was able to efficiently block uptake of [^18^F]olaparib in PSN-1 cells, lowering uptake to a 10 % of uptake in otherwise naïve cells (*P* < 0.0001). In contrast, while [^18^F]AZD2461 was efficiently taken up in PSN-1 cells and excess of non-radioactive AZD2461 was able to efficiently reduce uptake to 25 % of initial binding (*P* = 0.0022), the same excess of cold olaparib was only able to reduce uptake of [^18^F]AZD2461 to a mere 70 % of native binding (*P* = 0.024).

## Discussion

PARP enzymes play a crucial role in DNA damage repair. A wide range of highly selective and potent inhibitors of PARP have been developed (olaparib, rucaparib, talazoparib, veliparib, niraparib, and a plethora of others) and studied in clinical trials for their ability to compete with NAD^+^ binding as the PARP substrate. However, due to inhibition of the PARP3 isoform, expressed in a.o. bone marrow, may lead to haematological toxicity. Therefore, PARP inhibitors with different PARP inhibition profiles, such as AZD2461, with less ability to inhibit PARP-3, have also been evaluated as an alternative to compounds currently in clinical use.

In our previously reported study, we showed the strong potential of [^18^F]olaparib, a radio-isotopologue of the AstraZeneca compound olaparib, for PET imaging of PARP in a mouse model of human PDAC [17]. Other studies have demonstrated the ability of a range of radiolabelled PARP compounds to monitor PARP expression in a variety of preclinical cancer models [17–20]. However, among the 17-member PARP family, most studies focus on the implication of PARP1, but other isoforms are generally overlooked. As demonstrated by earlier reports [30], PARP inhibitors have different binding towards the 17 isoforms of PARP. Carney and co-workers described the use of a radiolabelled PARP binding agent (which catalytically inhibits, and therefore binds to, PARP1, 2 and, to a lesser extent, PARP3, 6, 7 and 8 and TNKS2). Hence, it is important to underline that the catalytic inhibition spectra for various PARP inhibitors (*e.g.* rucaparib, talazoparib, olaparib or AZD2461) are different and by extension their binding affinities to the various PARP isoforms. Our *in vitro* results show that our cell lines used have high PARP1 expression (Fig. 3c) and therefore represent good models to investigate AZD2461 for PET imaging of PARP expressing tumours.

^18^F-radiolabelling of neutral (hetero)arenes often requires a robust synthetic approach in order to secure radiosynthesis. Most studies rely on aliphatic radiolabelling or the use of radiolabelled prosthetic groups to produce radiotracers in reasonable activity yields (AY) and molar activities (*A*_m_) [21, 23]. However, as previously demonstrated with [^18^F]olaparib, in this study, we were able to efficiently radiolabel [^18^F]AZD2461 using the Cu-mediated radiofluorination method, originally developed by Tredwell et al. [31]. This process allows for the straightforward one-pot or two-step radiolabelling of a boronic ester precursor, for which retrosynthesis needs to be carefully planned. This method was shown to be translatable and automated, allowing high activity yield and molar activities (AY and *A*_m_ up to 4 % and 237 GBq/μmol, respectively, full details are displayed in Supplemental Information). One of the major side-products formed during reaction course is the undesired proto-deborylated compound 2 (Fig. 1) during ^18^F-fluorodeboronation of radiolabelling precursor 4. Our *in vitro* data showed that 2 is a potential inhibitor of PARP1, 2 and 3 isoforms with similar IC_50_ compared with [^19^F]AZD2461 1. This underlines the necessity of HPLC separation to avoid any contamination [17, 32]. With ^19^F references 1 and 2 in hand, we were able to demonstrate the efficient separation of both products by semi-preparative and analytical radio-HPLC, affording a difference in retention time of 3 min between both products. The final reformulated dose (between 100 and 600 MBq) were found suitable for following *in vitro* and *in vivo* experiments.

We then moved to investigate the ability of our radiotracer to target PARP expression *in vivo* in a range of tumour xenograft models of pancreatic cancer (Fig. 5). Comparing olaparib and AZD2461, highly similar metabolism and catabolism were observed, unsurprising given they possess very close chemical structures, yet bind differentially to the different PARP isoforms. This underlines the importance of using radiotracers to study structurally related inhibitors that bear similar physicochemical and elimination properties. Both [^18^F]olaparib and [^18^F]AZD2461 showed high tumour uptake in PDAC xenograft tissue. The specificity of the uptake of [^18^F]olaparib, demonstrated by the ability of an excess of cold olaparib to significantly block tumour uptake, but AZD2461 only able to block uptake of its radio-isotopologue to a lesser extent, led us to conclude that differences in target binding or specificity might be responsible. Follow-up *in vitro* experiments showed high uptake of both radiotracers, correlating with the high level of PARP expression in PSN-1 cells. Non-radioactive olaparib and AZD2461 were both able to reduce uptake of [^18^F]olaparib to a minimum of 10 %, corroborating the high degree of specific binding of olaparib, as demonstrated in our previous report [17]. While similarly high cell association was observed for [^18^F]AZD2461 in the same cell line, blocking showed a markedly reduced ability of olaparib to block cell uptake, contrary to an excess of AZD2461. While our previous *in vivo* experiments showed the lesser ability of non-radioactive AZD2461 to block [^18^F]AZD2461 tumour uptake compared with olaparib, variations in pharmacokinetics between the two inhibitors may be responsible for the differences observed. It is important to underline that there is no complete blocking with [^18^F]AZD2461 compared with [^18^F]olaparib using non-radioactive olaparib or AZD2461. Taken together, this suggests the ability of AZD2461 to bind to alternative targets in addition to those engaged by olaparib, although the precise targets have not been yet identified. While many studies describe the use of labelled inhibitors or associated variants [20, 23, 24, 27, 33], our study emphasises the critical evaluation of different PARP isoforms or alternative binding epitopes for PET imaging of PARP expression. Reilly and co-workers have shown the use of an ^18^F-labelled variant of AZD2461 [27]. However, differences in compound structures, PARP isoforms studied or model used make for a challenging direct comparison. When developing a DNA damage response PET imaging agent and in order to completely understand its target engagement, we hypothesise that non-PARP1 and PARP2 effects should also be considered to understand how tumour uptake of a radiolabelled compound relates to PARP biology. Conversely, any radiolabelled compound that only binds to PARP1 and 2 cannot be used to fully assess specific target engagement of an inhibitor also binding to, *e.g.* PARP3 or TNKS2. Even though, these results do not directly relate difference in specificity with PARP profile, we have shown that small variations in structure not known to be involved in PARP binding may lead to significant differences in binding selectivity and specificity *in vivo* and *in vitro;* these differences may underline the relative potential of the imaging agent as a diagnostic tool.

## Conclusion

Here, we have shown the ability of [^18^F]AZD2461 to target PARP expression in an *in vivo* mouse model of pancreatic cancer. While [^18^F]olaparib uptake was highly specific, we underline the difference in target binding with [^18^F]AZD2461 which shows its lesser ability to be utilised as a PARP imaging agent. As a consequence, PARP PET imaging, involving PARP radiolabelled inhibitors, requires knowledge of the binding profile and potential alternative targets in order to understand the biological information that can be obtained from these powerful imaging tools.

## Electronic supplementary material

ESM 1 (DOCX 1792 kb)

## References

[1] Lord CJ, Ashworth A (2012). The DNA damage response and cancer therapy. Nature..

[2] O'Connor MJ (2015). Targeting the DNA damage response in Cancer. Mol Cell.

[3] Murai J, Huang SY, Das BB (2012). Trapping of PARP1 and PARP2 by clinical PARP inhibitors. Cancer Res.

[4] Marchand JR, Carotti A, Passeri D (1844). Investigating the allosteric reverse signalling of PARP inhibitors with microsecond molecular dynamic simulations and fluorescence anisotropy. Biochim Biophys Acta.

[5] Lowery MA, Kelsen DP, Stadler ZK, Yu KH, Janjigian YY, Ludwig E, D'Adamo DR, Salo-Mullen E, Robson ME, Allen PJ, Kurtz RC, O'Reilly EM (2011). An emerging entity: pancreatic adenocarcinoma associated with a known BRCA mutation: clinical descriptors, treatment implications, and future directions. Oncologist..

[6] Patel AG, Sarkaria JN, Kaufmann SH (2011). Nonhomologous end joining drives poly(ADP-ribose) polymerase (PARP) inhibitor lethality in homologous recombination-deficient cells. Proc Natl Acad Sci U S A.

[7] Bryant HE, Schultz N, Thomas HD, Parker KM, Flower D, Lopez E, Kyle S, Meuth M, Curtin NJ, Helleday T (2005). Specific killing of BRCA2-deficient tumours with inhibitors of poly(ADP-ribose) polymerase. Nature..

[8] Gunderson CC, Moore KN (2015). Olaparib: an oral PARP-1 and PARP-2 inhibitor with promising activity in ovarian cancer. Future Oncol.

[9] Wang L, Wang Q, Xu Y, Han L (2019) Advances in the treatment of ovarian cancer using PARP inhibitors and the underlying mechanism of resistance. Curr Drug Targets 10.2174/1389450120666190925123507 31553293

[10] Livraghi L, Garber JE (2015). PARP inhibitors in the management of breast cancer: current data and future prospects. BMC Med.

[11] Rottenberg S, Jaspers JE, Kersbergen A, van der Burg E, Nygren AOH, Zander SAL, Derksen PWB, de Bruin M, Zevenhoven J, Lau A, Boulter R, Cranston A, O'Connor MJ, Martin NMB, Borst P, Jonkers J (2008). High sensitivity of BRCA1-deficient mammary tumors to the PARP inhibitor AZD2281 alone and in combination with platinum drugs. Proc Natl Acad Sci U S A.

[12] Hay T, Matthews JR, Pietzka L, Lau A, Cranston A, Nygren AOH, Douglas-Jones A, Smith GCM, Martin NMB, O’Connor M, Clarke AR (2009). Poly(ADP-ribose) polymerase-1 inhibitor treatment regresses autochthonous Brca2/p53-mutant mammary tumors in vivo and delays tumor relapse in combination with carboplatin. Cancer Res.

[13] Carney B, Kossatz S, Reiner T (2017). Molecular imaging of PARP. J Nucl Med.

[14] Knight JC, Koustoulidou S, Cornelissen B (2017). Imaging the DNA damage response with PET and SPECT. Eur J Nucl Med Mol Imaging.

[15] Pichler V, Berroteran-Infante N, Philippe C (2018). An overview of PET radiochemistry, part 1: the covalent labels (18)F, (11)C, and (13)N. J Nucl Med.

[16] Le Bars D (2006). Fluorine-18 and medical imaging: radiopharmaceuticals for positron emission tomography. J Fluor Chem.

[17] Wilson TC, Xavier MA, Knight J, Verhoog S, Torres JB, Mosley M, Hopkins SL, Wallington S, Allen PD, Kersemans V, Hueting R, Smart S, Gouverneur V, Cornelissen B (2019). PET imaging of PARP expression using (18)F-olaparib. J Nucl Med.

[18] Zhou D, Xu J, Mpoy C, Chu W, Kim SH, Li H, Rogers BE, Katzenellenbogen JA (2018). Preliminary evaluation of a novel (18)F-labeled PARP-1 ligand for PET imaging of PARP-1 expression in prostate cancer. Nucl Med Biol.

[19] Zmuda F, Blair A, Liuzzi MC, Malviya G, Chalmers AJ, Lewis D, Sutherland A, Pimlott SL (2018). An (18)F-labeled poly(ADP-ribose) polymerase positron emission tomography imaging agent. J Med Chem.

[20] Makvandi M, Pantel A, Schwartz L, Schubert E, Xu K, Hsieh CJ, Hou C, Kim H, Weng CC, Winters H, Doot R, Farwell MD, Pryma DA, Greenberg RA, Mankoff DA, Simpkins F, Mach RH, Lin LL (2018). A PET imaging agent for evaluating PARP-1 expression in ovarian cancer. J Clin Invest.

[21] Carney B, Carlucci G, Salinas B, di Gialleonardo V, Kossatz S, Vansteene A, Longo VA, Bolaender A, Chiosis G, Keshari KR, Weber WA, Reiner T (2016). Non-invasive PET imaging of PARP1 expression in glioblastoma models. Mol Imaging Biol.

[22] Schoder H, Demetriodesouza Franca P, Nakajima R, et al. PARP1/2 imaging with 18F-PARPi in patients with head and neck cancer. medRxiv*.* 2019:19009381

[23] Edmonds CE, Makvandi M, Lieberman BP, Xu K, Zeng C, Li S, Hou C, Lee H, Greenberg RA, Mankoff DA, Mach RH (2016). [(18)F]FluorThanatrace uptake as a marker of PARP1 expression and activity in breast cancer. American Journal of Nuclear Medicine and Molecular Imaging.

[24] Carlucci G, Carney B, Brand C, Kossatz S, Irwin CP, Carlin SD, Keliher EJ, Weber W, Reiner T (2015). Dual-modality optical/PET imaging of PARP1 in glioblastoma. Mol Imaging Biol.

[25] Reiner T, Lacy J, Keliher EJ, Yang KS, Ullal A, Kohler RH, Vinegoni C, Weissleder R (2012). Imaging therapeutic PARP inhibition in vivo through bioorthogonally developed companion imaging agents. Neoplasia..

[26] Oplustil O'Connor L, Rulten SL, Cranston AN, Odedra R, Brown H, Jaspers JE, Jones L, Knights C, Evers B, Ting A, Bradbury RH, Pajic M, Rottenberg S, Jonkers J, Rudge D, Martin NMB, Caldecott KW, Lau A, O'Connor MJ (2016). The PARP inhibitor AZD2461 provides insights into the role of PARP3 inhibition for both synthetic lethality and tolerability with chemotherapy in preclinical models. Cancer Res.

[27] Reilly SW, Puentes LN, Schmitz A, Hsieh CJ, Weng CC, Hou C, Li S, Kuo YM, Padakanti P, Lee H, Riad AA, Makvandi M, Mach RH (2019). Synthesis and evaluation of an AZD2461 [18F]PET probe in non-human primates reveals the PARP-1 inhibitor to be non-blood-brain barrier penetrant. Bioorg Chem.

[28] Guibbal F, Isenegger PG, Wilson TC, et al. (in press) Manual and automated Cu-mediated Radiosynthesis of the PARP inhibitor [18F]olaparib. Nat Protoc 10.1038/s41596-020-0295-7 32111986

[29] Taylor NJ, Emer E, Preshlock S, Schedler M, Tredwell M, Verhoog S, Mercier J, Genicot C, Gouverneur V (2017). Derisking the Cu-mediated (18)F-fluorination of heterocyclic positron emission tomography radioligands. J Am Chem Soc.

[30] Carney B, Kossatz S, Lok BH, Schneeberger V, Gangangari KK, Pillarsetty NVK, Weber WA, Rudin CM, Poirier JT, Reiner T (2018). Target engagement imaging of PARP inhibitors in small-cell lung cancer. Nat Commun.

[31] Tredwell M, Preshlock SM, Taylor NJ, Gruber S, Huiban M, Passchier J, Mercier J, Génicot C, Gouverneur V (2014). A general copper-mediated nucleophilic 18F fluorination of arenes. Angew Chem Int Ed Engl.

[32] Mossine AV, Brooks AF, Bernard-Gauthier V, Bailey JJ, Ichiishi N, Schirrmacher R, Sanford MS, Scott PJH (2018). Automated synthesis of PET radiotracers by copper-mediated (18) F-fluorination of organoborons: importance of the order of addition and competing protodeborylation. J Labelled Comp Radiopharm.

[33] Irwin CP, Portorreal Y, Brand C (2014). PARPi-FL--a fluorescent PARP1 inhibitor for glioblastoma imaging. Neoplasia (New York, NY).

