# Supplementary material for: [18F]AZD2461, an Insight on Difference in PARP Binding Profiles for DNA Damage Response PET Imaging
Source: Mol Imaging Biol. 2020 Apr 27;22(5):1226–34. doi: 10.1007/s11307-020-01497-6 (PMC7497465; doi:10.1007/s11307-020-01497-6)
Supplement: Supplementary file 1 — (DOCX 1792 kb) [file 11307_2020_1497_MOESM1_ESM.docx]

**Supplemental information for**

**[^18^F]AZD2461 for PET imaging of PARP**

Florian Guibbal,^1,2,^ † Samantha L. Hopkins,^1^ † Anna Pacelli,^1^ Patrick G. Isenegger,^2^ Michael Mosley,^1^ Julia Baguña Torres,^1^ Gemma Dias,^1^ Damien Mahaut,^2^ Rebekka Hueting,^1^ Véronique Gouverneur,*^2^ Bart Cornelissen*^1^

^1^ CRUK/MRC Oxford Institute for Radiation Oncology, Department of Oncology, University of Oxford, Oxford, UK

^2^ Department of Chemistry, University of Oxford, Oxford, UK

† These authors contributed equally to this study

*bart.cornelissen@oncology.ox.ac.uk, veronique.gouverneur@chem.ox.ac.uk

1. **Supplemental Material: Chemistry**

All chemicals were purchased from Acros, Alfa Aesar, Fluorochem, or Sigma-Aldrich and used as received without further purification. Solvents were purchased from Fisher, Rathburn or Sigma-Aldrich. Reactions were monitored by thin-layer chromatography (TLC) using Merck Kiesegel 60 F254 plates, silica gel column chromatography was performed over Merck silica gel C60 (40-60 μm). All NMR spectra were acquired using Bruker DPX200, AV400, AVB400, AVC500, AVB500 or DRX500 spectrometers. Proton and carbon-13 NMR spectra are reported as chemical shifts (δ) in parts per million (ppm) relative to the solvent peak using the Bruker internal referencing procedure (edlock). Fluorine-19 NMR spectra are referenced relative to CFCl_3_ in CDCl_3_. Coupling constants (J) are reported in units of hertz (Hz). The following abbreviations are used to describe multiplicities – s (singlet), d (doublet), t (triplet), q (quartet), m (multiplet), bs (broad singlet). High resolution mass spectra (HRMS, m/z) were recorded on a Bruker MicroTOF spectrometer using positive electrospray ionization (ESI^+)^ or on a Micromass GCT spectrometer using filed ionization (FI^+^) or chemical ionization (CI^+^). Infrared spectra were recorded either as the neat compound or in a solution using a Bruker Tensor 27 FT-IR spectrometer. Absorptions are reported in wavenumbers (cm^-1^) and only peaks of interest are reported. Melting points of solids were measured on a Griffin apparatus and are uncorrected. IUPAC names were obtained using the ACD/I-Lab service.

**Supplemental Figure S1.** Synthesis of 4-(3-(4-(cyclopropanecarbonyl)piperazine-1-carbonyl)-4-(4,4,5,5-tetra methyl-1,3,2-dioxaborolan-2-yl)benzyl)-2-((2-trimethlsilyl)ethoxy)methyl)phthalazin-1(*2H*)-one. *2-bromo-5-formylbenzonitrile was synthesised over two steps from 2-amino-5-bromobenzo-nitrile *via* formylation/Sandmeyer (51% over two steps), ***4-methoxypiperidine*.

**Supplemental Figure S2.** Synthesis of 4-(4-fluoro-3-(4-methoxypiperidine-1-carbonyl)benzyl)phthalazin-1(2H)-one.

**Supplemental Figure S3.** Synthesis of 4-(3-(4-methoxypiperidine-1-carbonyl)benzyl)phthalazin-1(2H)-one.


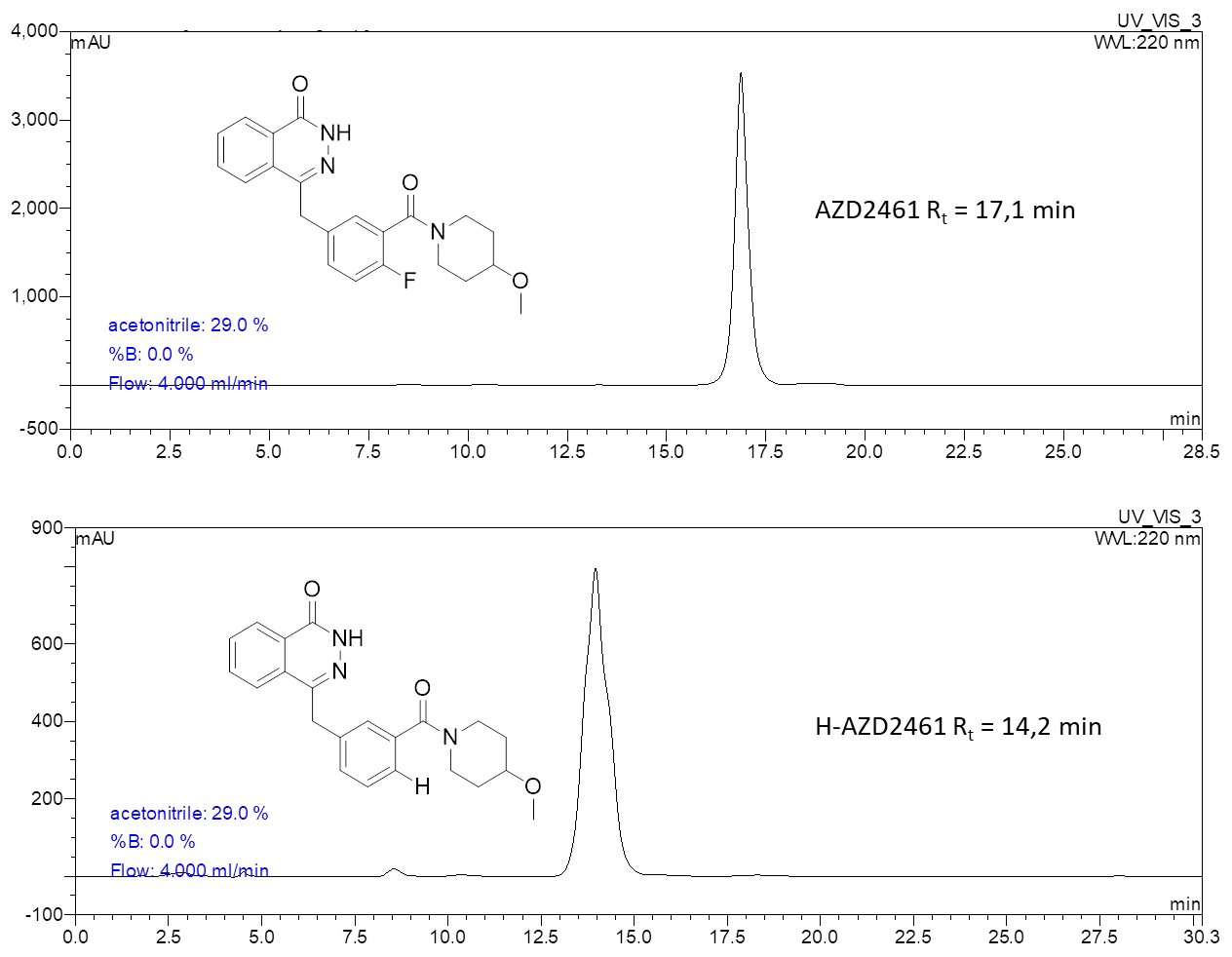


***Supplemental Figure S4.*** *HPLC UV traces of 4-(3-(4-methoxypiperidine-1-carbonyl)benzyl)phthalazin-1(2H)-one and AZD2461. HPLC Eluent: Synergi 4 µm Hydro-RP 80A, 250 x 10 mm with 29% MeCN/71% H_2_O (isocratic 4 mL/min) monitoring with UV (220 nm).*

**Dimethyl (3-oxo-1,3,-dihydroisobenzofuran-1-yl)phosphonate (as previously described [**[**1**](#_ENREF_1)**])**

Dimethylphosphite (0.92 mL, 10.0 mmol) was added dropwise to a solution of sodium (0.58 g, 10.8 mmol) in MeOH (15 mL) at 0°C. To the solution, 2-carboxybenzaldehyde (1.00 g, 6.66 mmol) was added portion-wise while stirring. The mixture was gradually warmed to room temperature and stirred for 6 hours. Methanesulfonic acid (0.77 mL, 11.9 mmol) was added dropwise and the mixture was stirred for another 30 minutes. The solution was concentrated in vacuo to produce a white solid, to which water was added (30 mL) and the crude product was extracted into DCM (3 × 30 mL). The organic layer was washed with water (2 × 30 mL), dried with MgSO_4_ and filtered. The filtrate was concentrated in vacuo and washed with Et_2_O (3 × 20 mL), affording Dimethyl-(3-oxo-1,3-dihydrobenzofuran-1-yl)phosphonate (1.48 g, 92%) as a white solid.

**^1^H NMR** (400 MHz, CD_3_OD) δ = 7.94 (d, *J* = 7.5 Hz, 1H), 7.83 (t, *J* = 7.5 Hz, 1H), 7.75 (d, *J* = 7.5 Hz, 1H), 7.68 (t, *J* = 7.5 Hz, 1H), 6.10 (d, *J* = 10.8, 1H), 3.91 (d, *J* = 10.8 Hz, 3H), 3.72 (d, *J* = 10.8 Hz, 3H). Data is in accordance with known literature [[1](#_ENREF_1)].

**2-Amino-5-formylbenzonitrile**

To a flame-dried round bottom flask was added 2-amino-5-bromo-benzonitrile (6.00 g, 30.5 mmol) and THF (180 mL). The solution was cooled to -78°C before *n*-BuLi in THF (2.5 M, 26.4 mL, 67 mmol) was added dropwise. The reaction was left to stir at -78°C for 2 h before quenching with DMF (6 mL, 77 mmol) and allowed to warm to room temperature. The solution was then extracted with DCM (3 x 100 mL) and washed with NaHCO_3_ (300 mL). The organic layers were collected, dried with MgSO_4_, and the excess solvent removed *in vacuo*. The crude material was then purified *via* flash column chromatography (*n-*Pent:EtOAc 8:2, R_f_ = 0.29) affording 2-amino-5-formylbenzonitrile (2.96 g, 66%) as a pale yellow solid.

**^1^H NMR** (400 MHz, CDCl_3_) δ = 9.75 (s, 1H), 7.92 (d*, J* = 1.8 Hz, 1H), 7.88 (dd, *J* = 8.6, 1.8 Hz, 1H), 6.83 (d, *J* = 8.6 Hz, 1H), 5.01 (bs, 2H). Data is in accordance with known literature [[1](#_ENREF_1)].

**2-Bromo-5-formylbenzonitrile**

To a round bottom flask containing 2-Amino-5-formylbenzonitrile (2.00 g, 13.7 mmol) at 0°C was added 6M HCl (13 mL) and fuming H_2_SO_4_ (13 mL). After being allowed to cool, a solution of sodium nitrite (1.98 g, 28.7 mmol) in H_2_O (5 mL) was added dropwise before allowing the reaction to stir for 30 minutes. The reaction mixture was then added dropwise to a solution of copper(II) bromide (4.28 g, 19.2 mmol) in 48% HBr (13 mL) at 0°C. The reaction was then stirred at 0°C for 60 min before being allowed to warm to room temperature and being stirred for another hour. Upon completion, the reaction was poured into an ice/water mixture before the organic layer was extracted with DCM (3 x 20 mL). The organic layer was then dried with MgSO_4_ and the excess solvent removed *in vacuo*. The crude material was purified by flash column chromatography (*n*-Pent:EtOAc 9:1, R_f_ = 0.35) to afford 2-bromo-5-formylbenzonitrile as a pale yellow solid (2.22 g, 77%).

**^1^H NMR** (400 MHz, CDCl_3_) δ = 10.00 (s, 1H), 8.15 (s*,* 1H), 7.95 (d, 8.4, 1H), 7.90 (d, *J* = 8.3 Hz, 1H). Data is in accordance with known literature [[1](#_ENREF_1)].

**2-Bromo-5-((3-oxoisobenzofuran-1(*3H*)-ylidene)methyl)benzonitrile**

A solution of dimethyl-(3-oxo-1,3-dihydrobenzofuran-1-yl)phosphonate (3.10 g, 12.7 mmol) and 2-bromo-5-formylbenzonitrile (2.22 g, 10.6 mmol) in THF (60 mL) was prepared at room temperature. The solution was cooled to 0°C followed by the addition of Et_3_N (2.94 mL, 21.1 mmol). The reaction mixture was warmed to room temperature and was stirred for 48 h, followed by concentration *in vacuo* to produce a white solid. The solid was suspended in water, collected by vacuum filtration and washed with hexane (2 × 30 mL) and Et_2_O (3 × 30 mL) affording 2-Bromo-5-((3-oxoisobenzofuran-1(*3H*)-ylidene)methyl)benzonitrile (3.32 g, 96%) as a white solid in an mixture of *e:z* stereoisomers (10:1) and a purity of 90% (water present). NMR spectra showed a 10:1 mixture of E and Z isomers.

**^1^H NMR** (400 MHz, DMSO-d_6_) δ = 8.20 – 8.07 (m, 2H, 2H*), 8.03 – 7.90 (m, 3H, 3H*), 7.84 – 7.49 (m, 2H, 2H*), 6.99 (s, 1H*), 6.98 (s, 1H) (where possible, shifts are assigned to each respective isomer). Data is in accordance with known literature [[1](#_ENREF_1)].

**2-Bromo-5-((4-oxo-3,4-dihydrophthalazin-1-yl)methyl)benzoic acid**

2-Bromo-5-[(3’-oxo-2’- benzofuran-1’-ylidene)methyl]benzonitrile (3.30 g, 10.1 mmol) was suspended in water (33 mL) and 9 M NaOH was added (6 mL, 90 mmol). The mixture was heated to 90°C and stirred for 24 h, after which it was warmed to reflux (140°C), followed by the addition of hydrazine monohydrate (6.2 mL, 200 mmol) and a further 48 h of stirring. The mixture was then cooled to room temperature and acidified with 6 M HCl to an approximate pH of 2. The solid precipitate was collected by vacuum filtration and washed with water (40 mL) and Et_2_O (3 × 20 mL) affording 2-Bromo-5-((4-oxo3,4-dihydrophthalazin-1-yl)methyl)benzoic acid (3.25 g, 91%) as a light yellow powder after drying under vacuum.

**^1^H NMR** (400 MHz, DMSO-d_6_) δ = 12.60 (s, 1H), 8.26 (dd, *J* = 7.7, 1.4 Hz, 1H), 7.98 – 7.95 (m, 1H), 7.90 (td, *J* = 7.9, 1.5 Hz, 1H), 7.83 (m, 1H), 7.71 (d, *J* = 2.3 Hz, 1H), 7.62 (d, *J* = 8.2 Hz, 1H), 7.36 (dd, *J* = 8.2, 2.3 Hz, 1H), 4.34 (s, 2H). Data is in accordance with known literature [[1](#_ENREF_1)].

**Methyl-2-bromo-5-((4-oxo-3,4-dihydrophthalazin-1-yl)methyl)benzoate**

To a solution of 2-Bromo-5-((4-oxo-3,4,-dihydrophthalazin-1-yl)methyl)benzoic acid (8.97 g, 25.0 mmol) in anhydrous DMF (75 mL) was added iodomethane (4.7 mL, 75 mmol) and anhydrous potassium carbonate (4.15 g, 30.0 mmol) in a flame dried flask. The reaction was then heated to 50°C and stirred overnight. Upon completion, the excess solvent was removed *in vacuo* and water (50 mL) was added to the crude material. The precipitate formed was filtered and washed with Et_2_O (3 x 40 mL) before drying *in vacuo* affording Methyl-2-bromo-5-((4-oxo-3,4-dihydrophthalazin-1-yl)methyl) benzoate as an off-white solid (7.46 g, 80%).

**^1^H NMR** (400 MHz, DMSO-d_6_) δ = 12.60 (s, 1H), 8.28 – 8.25 (m, 1H), 7.97-7.81 (m, 3H), 7.76-7.73 (m, 1H), 7.68-7.64 (m, 1H), 7.43-7.40 (m, 1H), 4.36 (s, 2H), 3.83 (s, 3H). Data is in accordance with known literature [[1](#_ENREF_1)].

**Methyl-2-bromo-5-((4-oxo-((2-(trimethylzilyl)ethoxy)methyl)-3,4-dihydro phthalazin-1-yl)methyl) benzoate**

To a round bottom flask under an atmosphere of argon containing Methyl-2-bromo-5-((4-oxo-((2-(trimethylzilyl)ethoxy)methyl)-3,4-dihydro phthalazin-1-yl)methyl) benzoate (7.04 g, 18.9 mmol) at 0°C was added anhydrous THF (125 mL) and sodium hydride (60% in dispersion oil, 1.51 g, 37.7 mmol). The reaction was stirred at 0°C for 30 min before warming to room temperature upon which 2-(trimethylsilyl)ethoxymethyl chloride (6.7 mL, 37.9 mmol) was added dropwise. The reaction was stirred overnight before the excess solvent was removed *in vacuo* and purified directly *via* flash column chromatography (*n*-Pent:EtOAc 8:2, R_f_ = 0.25) affording methyl-2-bromo-5-((4-oxo-((2-(trimethylzilyl)ethoxy)methyl)-3,4-dihydro phthalazine-1-yl)methyl)benzoate as a white solid (3.39 g, 36%).

**^1^H NMR** (400 MHz, CDCl_3_) δ = 8.50 ‒ 8.47 (m, 1H), 7.74 ‒ 7.72 (m, 3H), 7.63 ‒ 7.61 (m, 1H), 7.55 (d, *J* = 8.24, 1H), 7.23-7.20 (m, 1H), 5.59 (s, 2H), 4.28 (s, 2H), 3.92 (s, 3H), 3.78 ‒ 3.74 (m, 2H), 1.02 – 0.98 (m, 2H), 0.01 (s, 9H). Data is in accordance with known literature [[1](#_ENREF_1)].

**4-(3-(4-methoxypiperidine-1-carbonyl)-4-(4,4,5,5-tetramethyl-1,3,2-dioxaborolan-2-yl)benzyl)-2-((2-(trimethylsilyl)ethoxy)methyl)phthalazin-1(2H)-one**

^^

To a round bottom flask containing methyl-5-((4-oxo-3-((2-(trimethylsilyl)ethoxy) methyl)-4,4-dihydropphtalazin-1-yl) methyl)-2-(4,4,5,5-tetramethyl-1,3,2-dioxaborolan-2-yl)benzoate and bis(pinacolato) diboron (1.835g) was added THF (90 mL) before cooling to 0°C. Lithium hydroxide (2M solution in water, 8.0 mL, 16.0 mmol) was added dropwise at 0°C before stirring for 30 min at the same temperature. 1 M HCl was then added dropwise at 0°C until reaching pH 2. Aqueous NaCl (70 mL water + 20 mL brine) was added before extracting the resulting mixture with EtOAc (3 × 20 mL) and washing with brine (3 × 100 mL). The combined organic layers were dried over MgSO4, filtered and concentrated under reduced pressure. The crude was used as is for the next step without further purification.

The crude material was transferred to a round bottom flask upon which DCM (90 mL), HBTU (3.29 g, 8.67 mmol) and DIPEA (1.54 mL, 8.67 mmol) were added. A white suspension was obtained. After stirring at room temperature for 30 minutes, 4-methoxypiperidine (0.998g, 8.67 mmol) was added and the reaction mixture was stirred overnight. After stirring 16h, aqueous NaCl (70 mL water + 20 mL brine) was added to the resulting orange solution before extraction with DCM (3 × 20 mL). The combined organic layers were dried over MgSO_4_, filtered and concentrated under reduced pressure.

Purification by silica gel column chromatography using pentane:AcOEt 7:3 as eluant to recover the unreactive starting material then pure AcOEt. Further purification by HLPC afforded the title compound (40 mg, 15%) as a white powder.

**^1^H NMR** (400 MHz, CDCl_3_) δ = 8.48–8.43 (dd, 1H), 7.72 (d, *J* = 7.7 Hz, 1H), 7.70–7.65 (m, 2H), 7.65–7.59 (m, 1H), 7.26–7.21 (dd, 1H), 7.15 (d, *J* = 1.1 Hz, 1H), 5.59 (s, 2H), 4.32 (s, 2H), 4.04 (m, 1H), 3.83–3.70 (m, 2H), 3.43 (m, 2H), 3.34 (s, 3H), 3.29 (m, 1H), 2.93 (m, 1H), 1.94 (m, 1H), 1.69 (m, 2H), 1.48 (m, 1H), 1.28 (s, 12H), 1.08–0.93 (m, 2H), 0.00 (s, 9H).**^13^C NMR** (100 MHz, CDCl_3_) δ = 170.5, 160.0, 144.7, 143.6, 140.9, 136.1, 133.2, 131.3, 129.2, 128.3, 127.7, 127.5, 125.3, 125.2, 83.9 (2C), 78.9, 77.0, 75.5, 67.1, 55.6, 44.3, 39.0, 38.6, 29.5, 24.8 (4C), 18.0, -1.46 (3C) (the carbon bearing the boron substituent is not observed); **IR** (*ν,* cm^-1^): 2950, 2563, 2361, 2160, 2022, 1976, 1663, 1637, 1444, 1351, 1319, 1145, 1093, 1025, 857, 835, 746; **HRMS** (ESI) for C_34_H_49_N_3_O_6_^10^B^28^Si [M+Na]^+^ requires 634.34831 found 634.34784; **Mp**: 74°C.

**3-((4-oxo-3,4-dihydrophthalazin-1-yl)methyl)benzoic acid**

A solution of Dimethyl-(3-oxo-1,3-dihydrobenzofuran-1-yl)phosphonate (7.25 g, 30.0 mmol) and 3-formylbenzonitrile (2.62 g, 20.0 mmol) in THF (250 mL) was prepared at room temperature. The solution was then cooled to 0°C followed by the addition of Et_3_N (6.10 mL, 30.0 mmol). The reaction mixture was warmed to room temperature and was stirred for 48 h, followed by concentration *in vacuo* to produce a white solid. The solid was suspended in water, collected by vacuum filtration and washed with hexane (2 × 20 mL) and Et_2_O (3 × 20 mL) affording 3-((3-oxoisobenzofuran-1(3*H*)-ylidene)methyl)benzonitrile (4.40 g) as a crude white solid which was then taken through to the next step without further purification. 3-((3-oxoisobenzofuran-1(3*H*)-ylidene)methyl)benzonitrile (4.40 g, 17.8 mmol) was suspended in water (100 mL) and 13 M NaOH was added (26.4 mL, 238 mmol). The mixture was heated to 90°C and stirred for 2 h, after which it was warmed to reflux (140°C), followed by the addition of hydrazine monohydrate (7.48 mL, 240 mmol) and a further 16 h of stirring. The mixture was then cooled to room temperature and acidified with 5 M HCl to an approximate pH of 2. The solid precipitate was collected by vacuum filtration and washed with water (100 mL) and Et_2_O (3 × 100 mL) affording 3-((4-oxo-3,4-dihydrophthalazin-1-yl)methyl)benzoic acid (4.18 g, 15.0 mmol, 50%, two steps) as a white solid.

**^1^H NMR** (400 MHz, DMSO-*d_6_*) δ = 12.64 (s, 1H), 8.25 (dd, *J* = 7.9, 1.4 Hz, 1H), 7.96 (d, *J* = 7.9 Hz, 1H), 7.91 – 7.85 (m, 2H), 7.82 (dd, *J* = 7.6, 1.2 Hz, 1H), 7.80 – 7.75 (m, 1H), 7.58 (dt, *J* = 7.7, 1.5 Hz, 1H), 7.42 (t, *J* = 7.7 Hz, 1H), 4.38 (s, 2H). Data is in accordance with known literature [[1](#_ENREF_1)].

**4-(3-(4-methoxypiperidine-1-carbonyl)benzyl)phthalazin-1(2H)-one**

To a solution of 3-((4-oxo-3,4-dihydrophthalazin-1-yl)methyl)benzoic acid (50 mg, 0.18 mmol) in DMA (2 mL) was added DIPEA (61 μL, 0.36 mmol) and HBTU (75 mg, 0.20 mmol). The reaction mixture was stirred for 1 h before addition of 4-methoxy piperidine (21 mg, 0.18 mmol) was carried out. The reaction mixture was then stirred at room temperature for 48 h, and the reaction mixture was extracted with DCM (3 × 10 mL) and washed with water (3 × 10 mL). The organic layers were collected, dried with MgSO_4_ and the excess solvent removed *in vacuo*. Purification *via* reverse phase HLPC was then carried out affording 4-(3-(4-methoxypiperidine-1-carbonyl)benzyl)phthalazin-1(2H)-one (37 mg, 54**%**) as a white solid.

**^1^H NMR** (400 MHz, CDCl_3_) δ = 11.81 (bs, 1H), 8.43 – 8.39 (m, 1H), 7.69 – 7.65 (m, 3H), 7.27 – 7.25 (m, 3H), 7.20 – 7.18 (m, 1H), 4.26 (s, 2H), 3.99 – 3.88 (m, 1H), 3.46 – 3.36 (m, 3H), 3.29 (s, 3H), 3.11 – 3.03 (m, 1H), 1.93 – 1.79 (m, 1H), 1.69 – 1.54 (m, 2H), 1.50 – 1.36 (m, 1H); **^13^C NMR** (101 MHz, CDCl_3_) δ = 170.1, 160.7, 146.1, 138.2, 136.7, 133.7, 131.6, 129.8 (2C), 129.1, 128.4, 127.2, 127.0, 125.4, 125.3, 125.1, 75.4, 55.9, 44.4, 39.4, 38.8, 31.4; **HRMS** (ESI) for C_22_H_24_N_3_O_3_ [M+H]^+^ requires 378.1812 found 378.1809. **IR** (ν, cm-1): 3181, 2930, 2517, 2160, 2024, 1978, 1655, 1624, 1443, 1260, 1092, 1023, 790, 772. **MP**: 79°C.

**2-Fluoro-5-((3-oxoisobenzofuran-1(*3H*)-ylidene)methyl)benzonitrile**

A solution of Dimethyldimethyl-(3-oxo-1,3-dihydrobenzofuran-1-yl)phosphonate (1.00 g, 4.13 mmol) and 2-fluoro-5-formylbenzonitrile (0.62 g, 4.13 mmol) in THF (50 mL) was prepared at room temperature. The solution was then cooled to 0°C followed by the addition of Et_3_N (0.69 mL, 4.96 mmol). The reaction mixture was allowed to warm up to room temperature and was stirred for 48 h, followed by concentration *in vacuo* to produce a white solid. The solid was suspended in water, collected by vacuum filtration and washed with hexane (2 × 20 mL), Et_2_O (2 × 20 mL), and MeOH (2 × 20 mL) affording 2-Fluoro-5-((3-oxoisobenzofuran-1(*3H*)-ylidene)methyl)benzonitrile (0.89 g, 90%) as a white solid.

NMR spectra showed a 3:1 mixture of E and Z isomers. Where possible, shifts are assigned to each respective isomer. **^1^H NMR** (400 MHz, DMSO-*d_6_*) δ = 8.21 ‒ 8.12 (m, 1H), 8.08 (dt, *J* = 8.0, 1.0 Hz, 1H), 8.00 ‒ 7.97 (m, 1H), 7.92 (t, *J* = 7.6 Hz, 1H), 7.75 ‒ 7.67 (m, 1H), 7.65 (t, *J =* 9.0 Hz, 1H), 6.98 (s, 1H). Data is in accordance with known literature [[1](#_ENREF_1)].

**2-Fluoro-5-((4-oxo-3,4,-dihydrophthalazin-1-yl)methyl)benzoic acid**

2-Fluoro-5-[(3’-oxo-2’- benzofuran-1’-ylidene)methyl]benzonitrile (0.50 g, 1.89 mmol) was suspended in water (3 mL) and 13 M NaOH was added (0.67 mL). The mixture was heated to 90°C and stirred for 24 h, after which it was cooled to 70°C, followed by the addition of hydrazine monohydrate (1.34 mL, 26.9 mmol) and a further 72 h of stirring. The mixture was then cooled to room temperature and acidified with 8 M HCl to an approximate pH of 4. The solid precipitate was collected by vacuum filtration and washed with water (3 × 25 mL) and Et_2_O (4 × 25 mL) affording 2-Fluoro-5-((4-oxo3,4-dihydrophthalazin-1-yl)methyl)benzoic acid (0.536 g, 95%) as a red solid [[1](#_ENREF_1)].

**^1^H NMR** (400 MHz, DMSO-*d_6_*) δ = 12.57 (s, 1H), 8.26 (dd, *J* = 7.8, 0.8 Hz, 1H), 7.98 (d, *J* = 7.9 Hz, 1H), 7.93 ‒ 7.86 (m, 1H), 7.86 ‒ 7.79 (m, 2H), 7.61 ‒ 7.54 (m, 1H), 7.23 (dd, *J* = 10.8, 8.5 Hz, 1H), 4.35 (s, 2H); **{^1^H} ^19^F NMR** (376 MHz, DMSO-*d_6_*) δ = -114.0. Data is in accordance with known literature [[1](#_ENREF_1)].

**4-(4-fluoro-3-(4-methoxypiperidine-1-carbonyl)benzyl)phthalazin-1(2H)-one (AZD2461)**

To a solution of 2-Fluoro-5-((4-oxo3,4-dihydrophthalazin-1-yl)methyl)benzoic acid (100 mg, 0.34 mmol) in DMA (2 mL) was added DIPEA (114 *μ*L, 0.67 mmol) and HBTU (140 mg, 0.37 mmol). The reaction mixture was stirred for 1 hour before addition of 4-methoxy piperidine (39 mg, 0.34 mmol) was carried out. The reaction mixture was stirred at room temperature for 48 h. The reaction mixture was then extracted with DCM (3 × 10 mL) and washed with water (3 × 10 mL). The organic layers were collected, dried with MgSO_4_ and the excess solvent removed *in vacuo*. Purification *via* reverse phase HLPC was carried out affording 4-(4-fluoro-3-(4-methoxypiperidine-1-carbonyl)benzyl)phthalazin-1(2H)-one (AZD2461) (60 mg, 44**%**) as a white solid.

**^1^H NMR** (400 MHz, CDCl_3_) δ = 11.03 (s, 1H), 8.48 ‒ 8.46 (m, 1H), 7.78 ‒ 7.70 (m, 3H), 7.33 ‒ 7.25 (m, 2H), 7.00 (t, *J* = 8.0 Hz, 1H), 4.28 (s, 2H), 4.06 ‒ 3.90 (m, 1H), 3.65 – 3.36 (m, 3H), 3.35 (s, 3H), 3.20 – 3.09 (m, 1H), 1.95 ‒ 1.90 (m, 1H), 1.73 – 1.66 (m, 2H), 1.55 ‒ 1.49 (m, 1H); **^13^C NMR** (101 MHz, CDCl_3_) δ = 164.9, 160.8, 157.2 (d, *J_CF_* = 240 Hz), 145.8, 134.2, 133.8, 131.7, 131.2 (d, *J_CF_* = 8 Hz), 129.7, 129.0 (d, *J_CF_* = 4 Hz), 128.4, 127.3, 125.3, 124.6 (d, *J_CF_* = 19 Hz), 116.2 (d, *J_CF_* = , 22 Hz), 75.2, 55.9, 44.3, 39.0, 37.9, 31.2, 30.1; **{^1^H}^19^F NMR** (376 MHz, CDCl_3_) δ = - 117.9 (m); **HRMS** (ESI) for C_22_H_23_N_3_O_3_F_1_ [M+H]^+^ requires 396.1718 found 396.1712. **IR** (ν, cm-1): 3184, 2929, 2161, 2033, 1630, 1447, 1226, 1099, 1087, 804, 788, 772. **MP**: 85°C.

**Synthesis of [Cu(OTf)_2_(impy)_4_] complex**

The copper complex was prepared as previously described [[1](#_ENREF_1)].

1. **Radiochemical synthesis**

**Radiochemistry:**

**Procedure for the manual radiosynthesis and isolation of [^18^F]AZD2461:**

[^18^F]Fluoride was produced by Alliance Medical (UK) via the ^18^O(p,n)^18^F reaction and delivered as [^18^F]fluoride in ^18^O-enriched-water. Radiosynthesis, azeotropic drying, radiolabelling and purification were performed on a NanoTek microfluidic device (Advion).

[^18^F]AZD2461 was obtained *via* the Cu-mediated ^18^F-fluorodeboronation of the corresponding boronic ester precursor using methodology previously described by Wilson *et al* [[1](#_ENREF_1)]. [^18^F]Fluoride was separated from ^18^O-enriched-water using an anion exchange cartridge (Sep-Pak Accell Plus QMA Carbonate Plus Light Cartridge, 46 mg Sorbent per Cartridge, 40 µm particle size, Waters, preconditioned with 10 mL of H_2_O) and released with 900 μL (in 6 x 150 µL portions) of a solution of K_222_/K_2_C_2_O_4_/K_2_CO_3_ (Kryptofix®222 (6.3 mg), K_2_C_2_O_4_ (1 mg) and K_2_CO_3_ (0.1 mg) in 1 mL of MeCN/H_2_O, 4:1) into a 5 mL V-vial containing a magnetic stir bar in the concentrator. The solution was dried with five cycles of azeotropic drying with MeCN (5 x 200 μL, additional MeCN was added 2 x 500 μL if better drying was needed) under a flow of N_2_ at 100-105°C. The 5 mL vial containing the dried [^18^F]KF/K_2.2.2_ complex was purged with 20 mL of air using a syringe and then a solution of arylboronate precursor (12.7 mg, 0.02 mmol) and Cu(OTf)_2_(impy)_4_ (19 mg, 0.03 mmol) in anhydrous 1,3-dimethyl-2-imidazolidinone (DMI) (300 μL) was added. The mixture was heated at 120°C for 20 min in a sealed vial with stirring. After 20 min, TFA (370 μL) was added and stirring was continued at 125°C for further 20 min. The reaction was then cooled to room temperature before quenching with H_2_O (6 mL) and the solution obtained was eluted over an Oasis HLB Plus cartridge (preconditioned with 2 mL MeOH and 10 mL H_2_O). The vial was then rinsed with CH_3_CN:H_2_O (1:9, 2.0 mL), and the solution was eluted over the Oasis HLB cartridge after which the product was recovered from the cartridge by elution with CH_3_CN (2.0 mL) into a new 5 mL V-vial. The MeCN was evaporated under a flow of N_2_ at 100°C until approximately 50-100 µL remained. The reaction mixture was then diluted in with 33% MeCN/ 67% 25 mM NH_4_HCO_2_ in H_2_O and loaded directly onto a 2 mL HPLC loop and injected on a semi-Prep HPLC column (Synergi 4 µm Hydro-RP 250x10mm) and eluted into a collection vial with 33% MeCN/ 67% 25 mM NH_4_HCO_2_ in H_2_O monitoring with UV (254 nm) and radioactive traces.

The [^18^F]AZD2461 was collected in 18 mL of H_2_O and eluted over an Oasis HLB Plus cartridge (preconditioned with 2 mL MeOH and 10 mL H_2_O). The cartridge was washed with H_2_O (1.0 mL), after which the product was eluted with EtOH (2.0 mL). The ethanol was evaporated under a flow of N_2_ while heating at 100°C. The dry residue was then re-dissolved in 10% DMSO/PBS (pH = 7.4).

The molar activity of [^18^F]AZD2461 was assessed by radio-HPLC, using an analytical Synergi 4 µm Hydro-RP 80A column, 150 x 4.6 mm eluted with 29% MeCN/71% H_2_O (isocratic 1 mL/min), monitoring with UV (220 nm) and radioactive traces.

Molar activity was calculated based on a standard series of non-radioactive reference AZD2461 (Supplemental Figure S7).


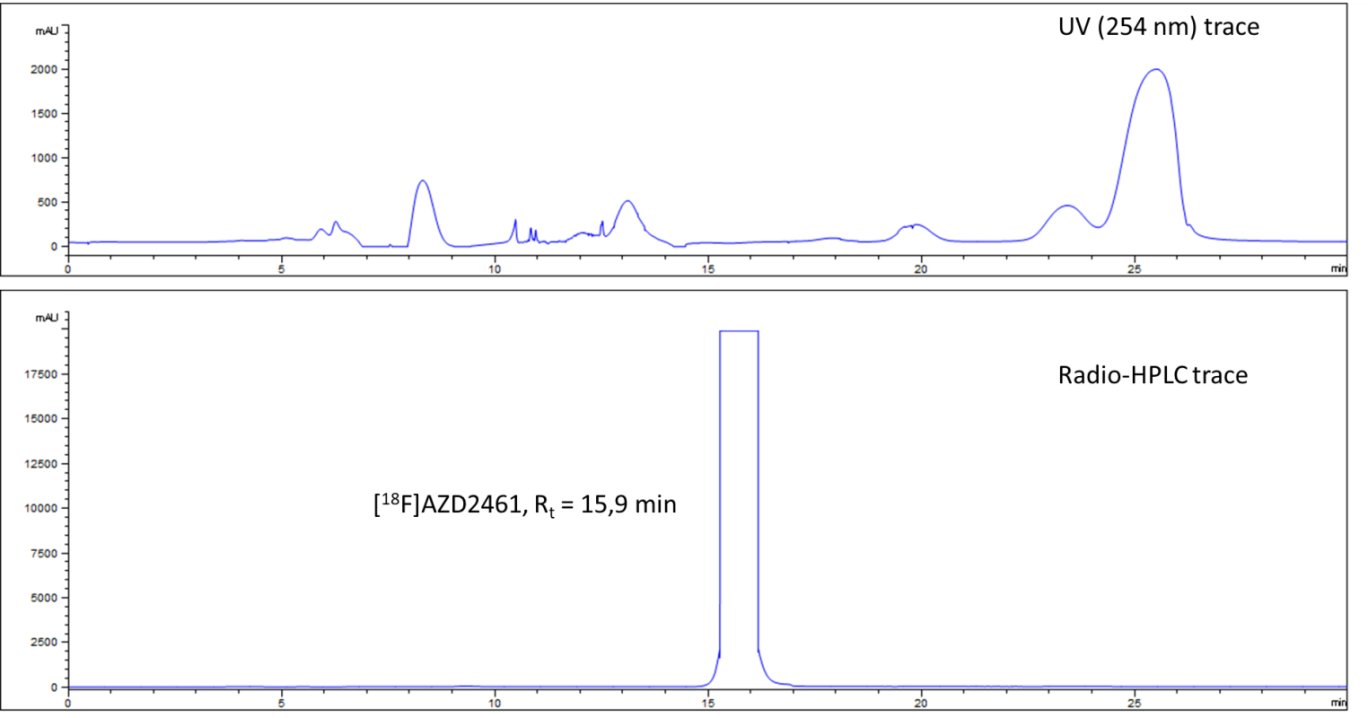


**Supplemental Figure S5.** Semi-prep radioHPLC purification of [^18^F]AZD2461 manual radiolabelling


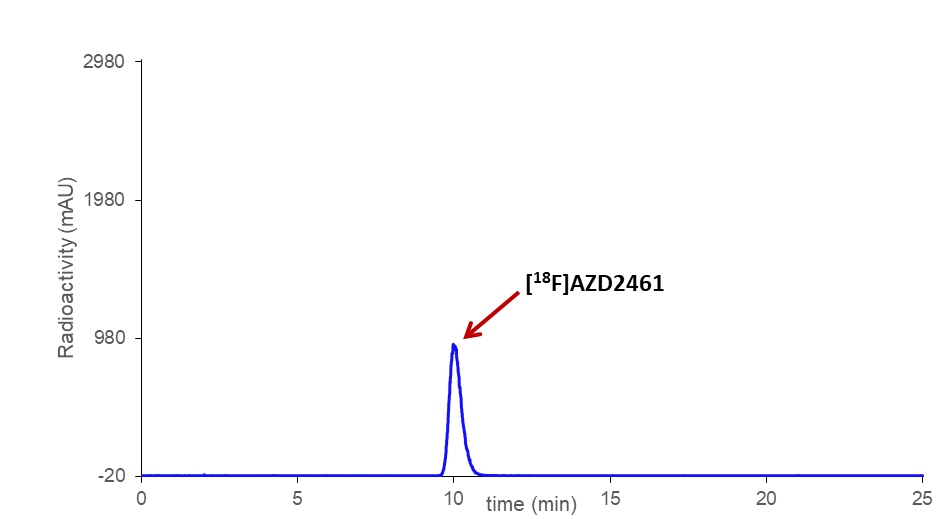


**(A) Radio trace of [^18^F]AZD2461**


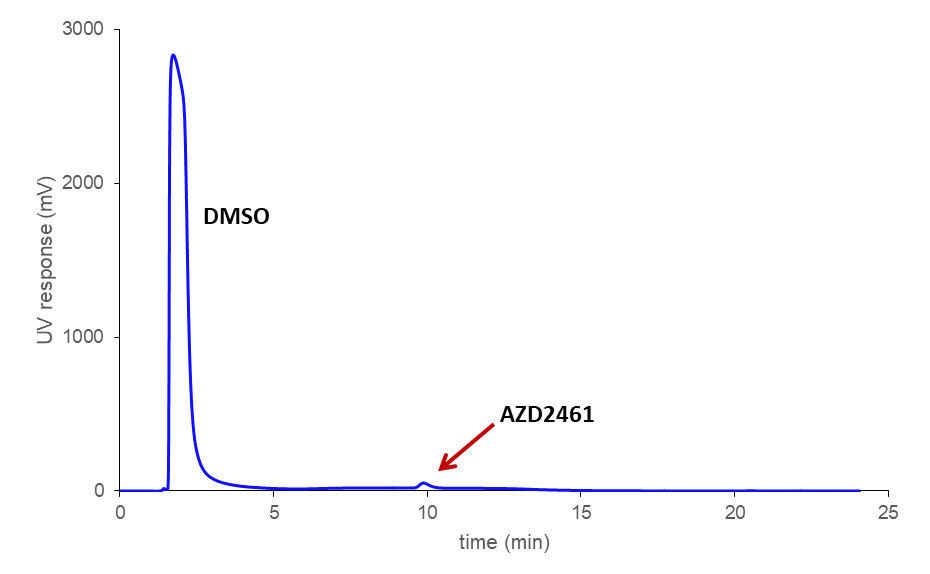


**(B) UV trace of [^18^F]AZD2461**


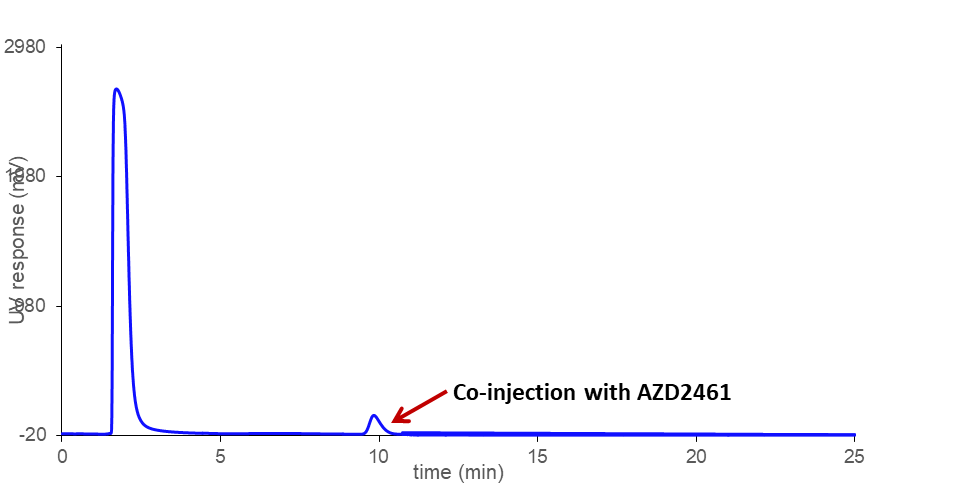


**(C) Co-injection of [^18^F]AZD2461 and reference**

**Supplemental Figure S6.** Quality control analysis of [^18^F]AZD2461. (A) Radio trace; (B) UV trace; (C) Co-injection with authentic cold reference.

**Procedure for the automated radiosynthesis and isolation of [^18^F]AZD2461:**

The automated radiosynthesis of [^18^F]AZD2461 was performed in accordance with a similar protocol to our previous reports [[2](#_ENREF_2)].

Briefly, aqueous [^18^F]fluoride (typically 10-20 GBq, PETIC Cardiff) was delivered to an Eckert & Ziegler Modular-Lab platform. [^18^F]fluoride was trapped on a QMA carbonate cartridge, which was previously conditioned with 5 mL of water for injection, and released into a reactor with 1 mL of a solution containing 6.3 mg of Kryptofix®222 in 800 µL of MeCN, 1 mg of K_2_C_2_O_4_ in 100 µL and 0.1 mg K_2_CO_3_ in 100 µL. The reactor was heated to 105°C and the water was azeotropically dried under a N_2_ flow with the addition of 3 × 500 µL of dry MeCN. Air was introduced into the reaction vial through a valve open to the hot cell *via* a vacuum pump for 30 seconds.

[Cu(OTf)_2_(impy)_4_] and BPin precursor, which were previously dissolved in two separate vials in 300 µL of DMI each, were then added to the reaction vial. The reactor was heated at 120 °C to perform the labelling. Every 5 minutes, more air was added to the reaction vial for 10 seconds. After 20 minutes, 500 µL of TFA were added and the temperature was increased to 125 °C to perform the deprotection.

After 20 minutes, the reaction mixture was quenched with 900 µL of water and cooled down to 30 °C. The mixture was transferred through a fritted reservoir to a vial containing 1 mL of ammonium formate 25 mM. The empty reactor was washed with 1 mL of 9:1 water:MeCN and this solution was transferred to the same vial. The 4 mL solution thus obtained was injected onto a Synergi 4 µM Hydro-RP 80 Å 250 × 10 mm semi-preparative column, which was previously equilibrated with 25% MeCN in ammonium formate 25 mM. The peak corresponding to [^18^F]AZD2461 was collected into a glass bottle containing 80 mL of water. This solution was transferred to a C18 light cartridge, which was previously conditioned with 10 mL EtOH followed by 10 mL of water, to trap [^18^F]AZD2461. The cartridge was washed with 1 mL water and [^18^F]AZD2461 was eluted from the cartridge using 1 mL of EtOH into a vial containing 9 mL of saline to yield a final reformulation of 10% EtOH in saline. 20 µL of this dose was used to perform the quality control. The sample was injected onto a Synergi 4 µm Hydro-RP 80 Å 150 × 4.6 mm analytical column and eluted with 29% MeCN in ammonium formate 28.5 mM in isocratic conditions. A co-injection of the dose with a sample of the cold reference confirmed the identity of the radiotracer.


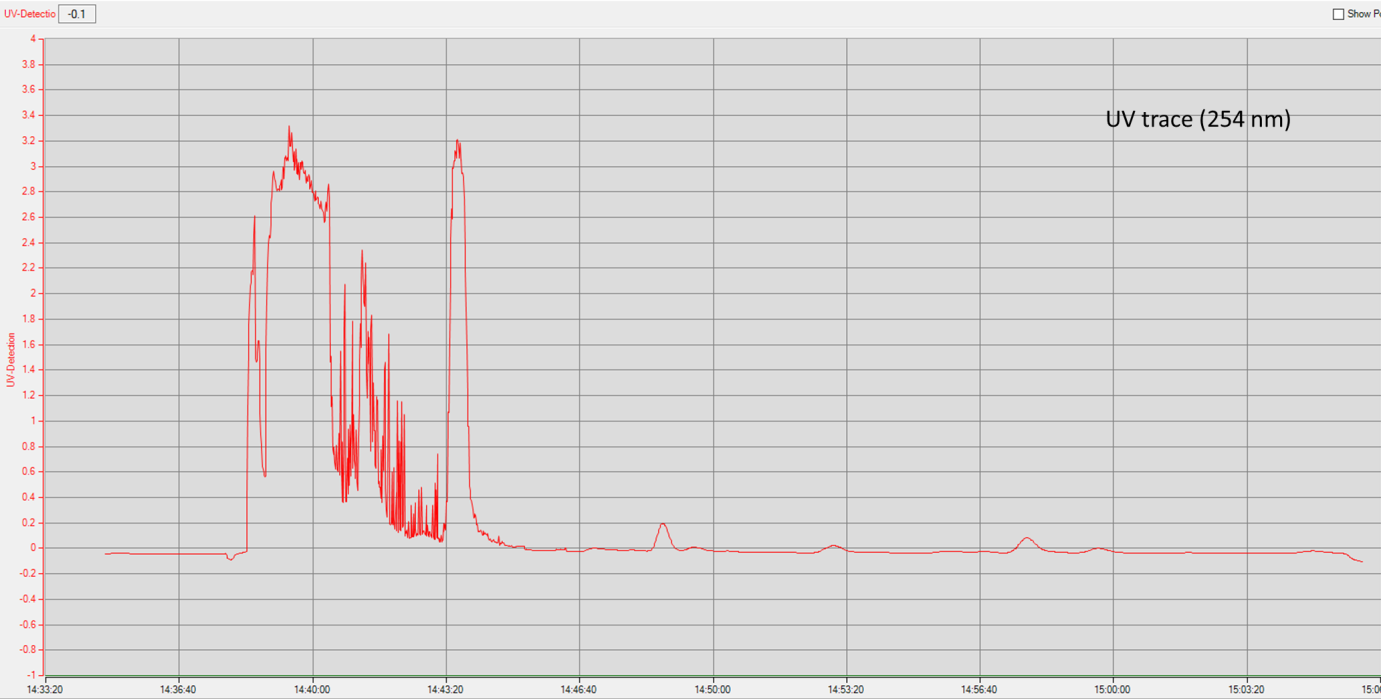

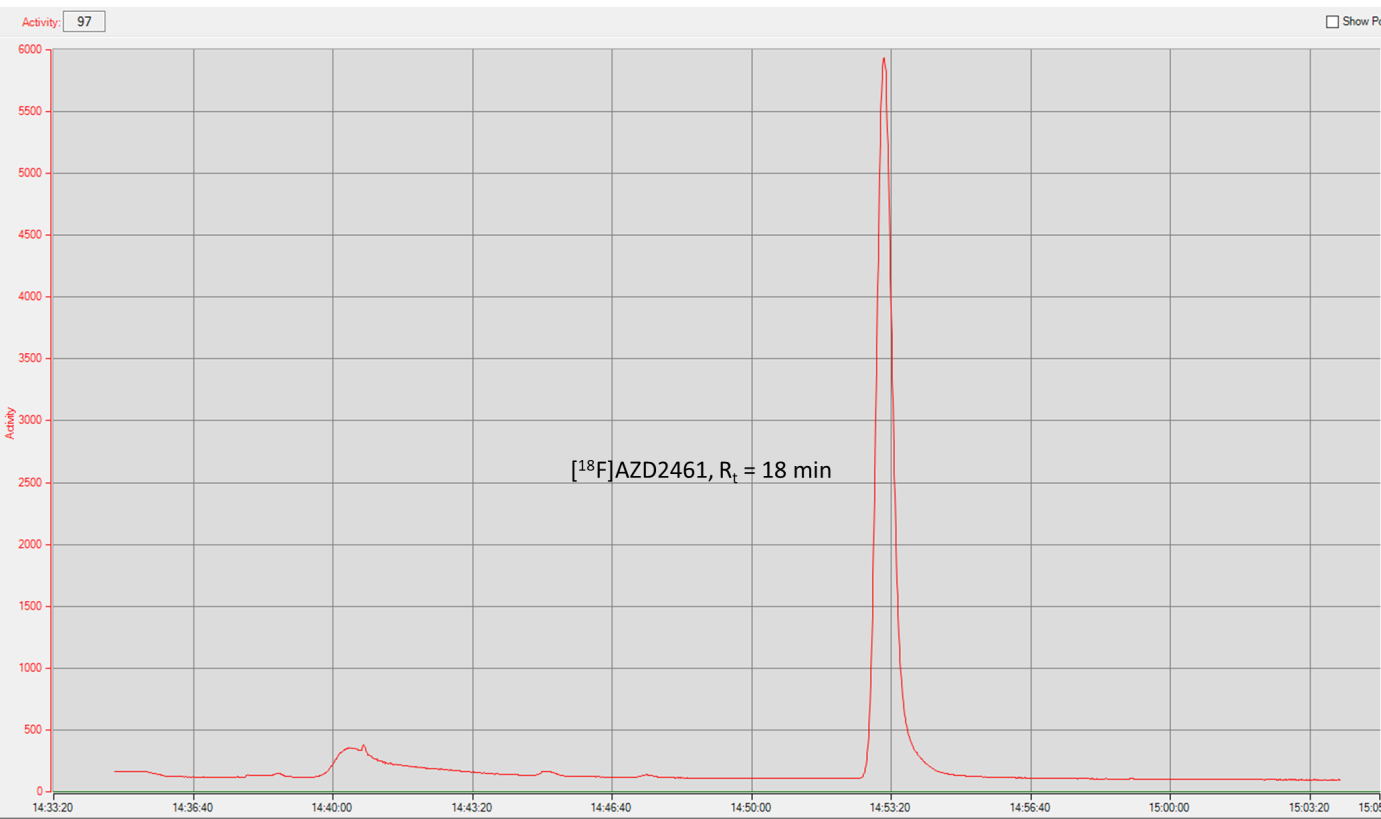


**Supplemental Figure S7.** Semi-preparative radioHPLC purification of [^18^F]AZD2461 automated radiolabelling.


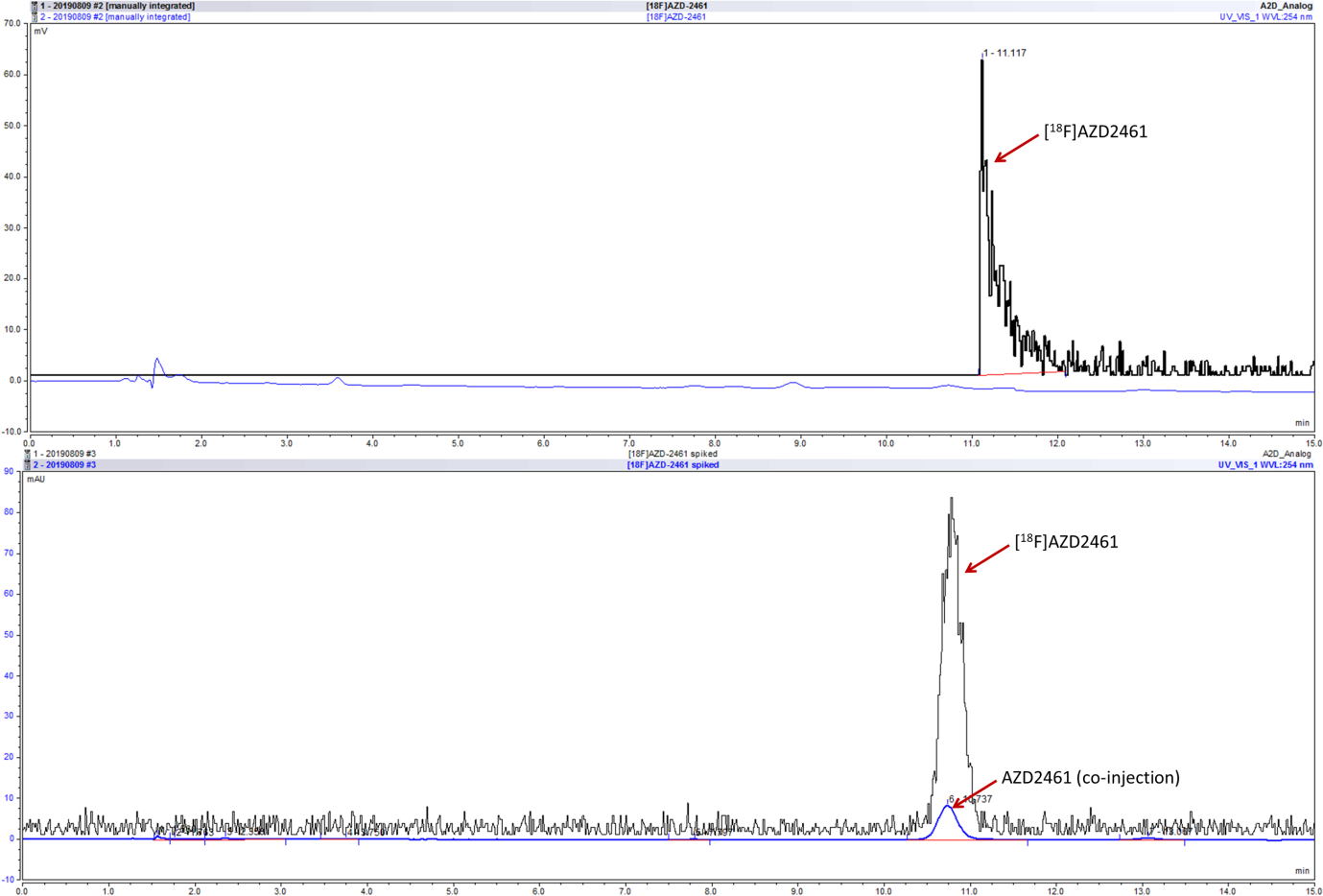


**Supplemental Figure S8.** Quality control analysis of [^18^F]AZD2461. Radio trace; UV trace (254 nm); Co-injection with authentic cold reference.


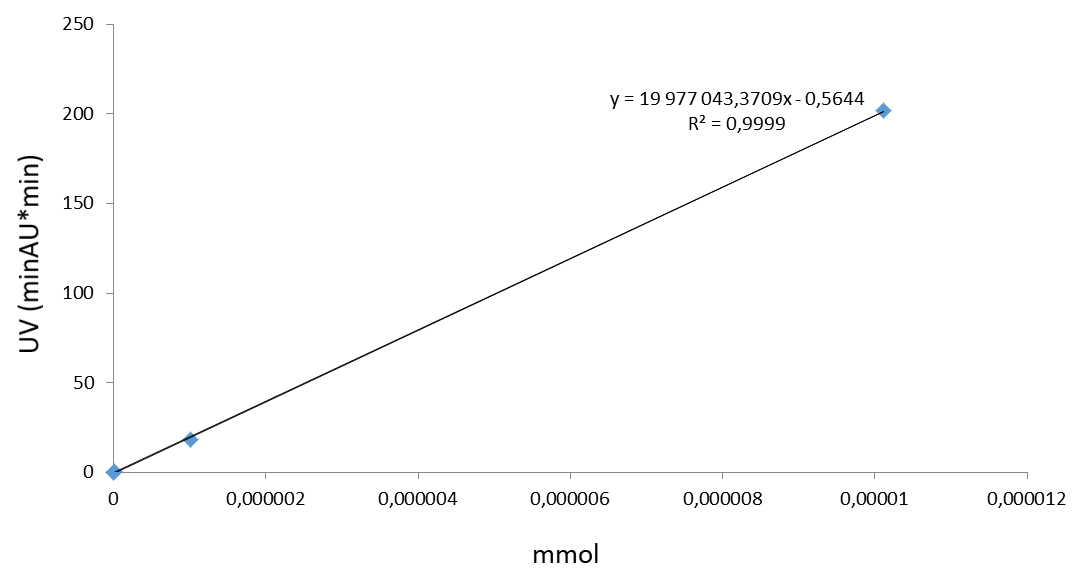


**Supplemental Figure S9.** Calibration curve for molar activity determination of [^18^F]AZD2461.

1. **NMR Spectra of Novel Compounds:**

**4-(3-(4-methoxypiperidine-1-carbonyl)-4-(4,4,5,5-tetramethyl-1,3,2-dioxaborolan-2-yl)benzyl)-2-((2-(trimethylsilyl)ethoxy)methyl)phthalazin-1(2H)-one**

^^
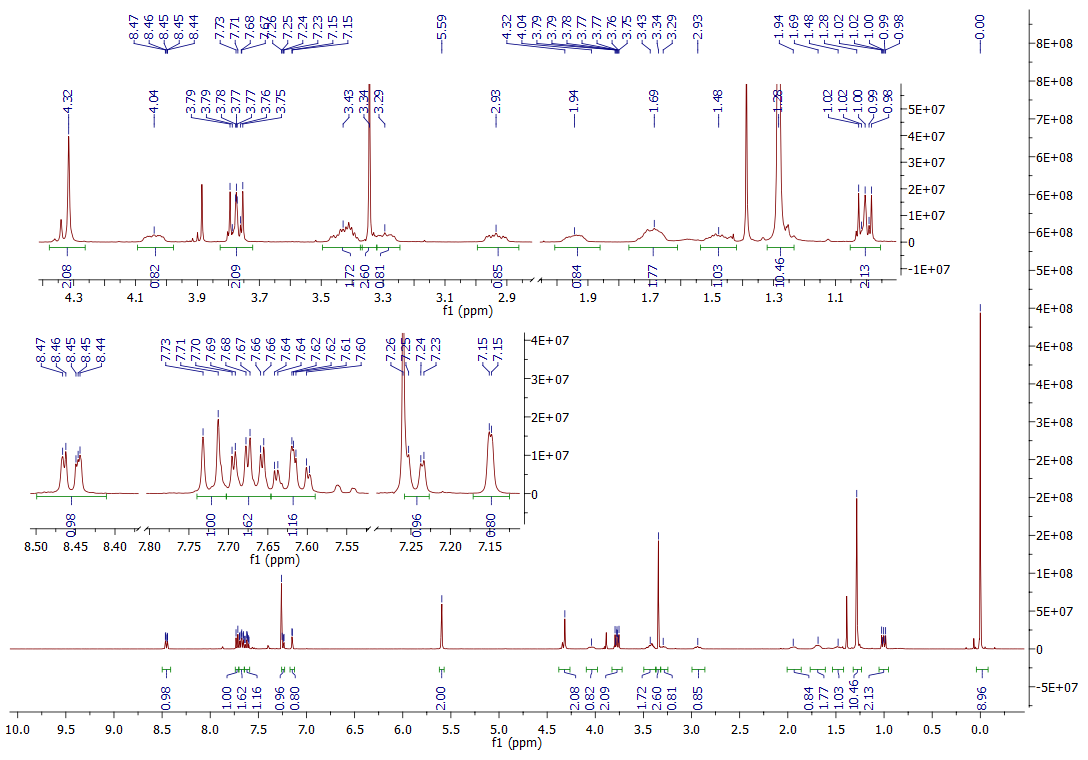


^^
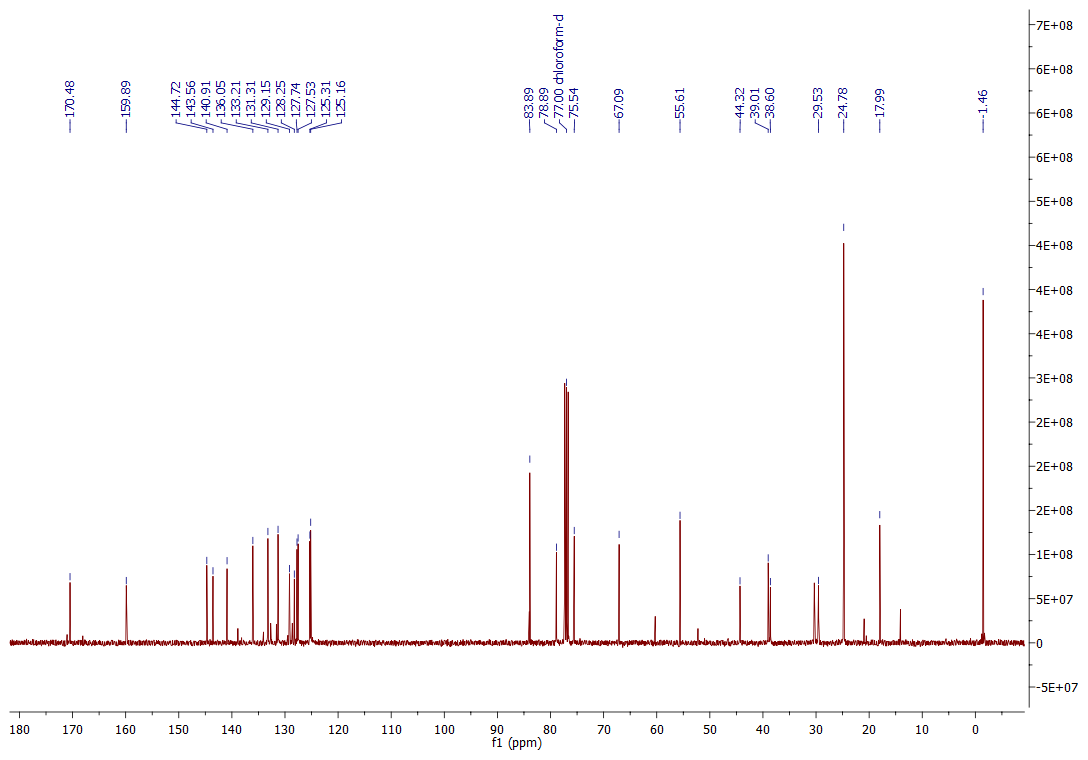


**4-(3-(4-methoxypiperidine-1-carbonyl)benzyl)phthalazin-1(2H)-one**

**
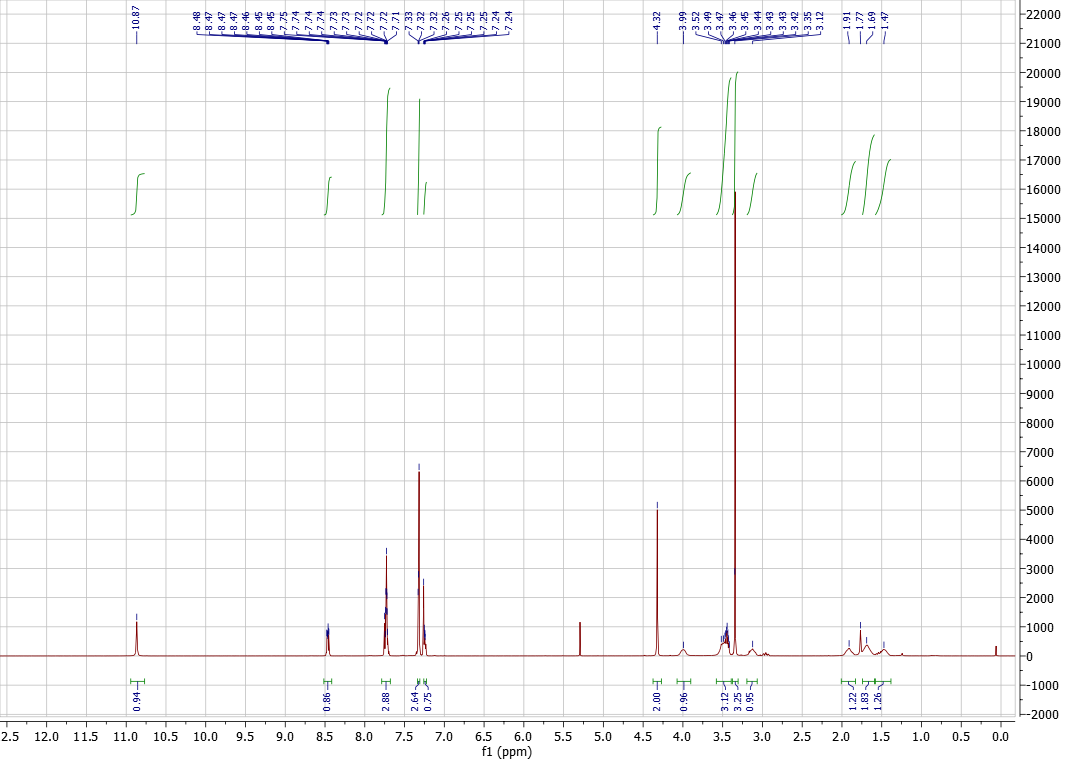
**

**
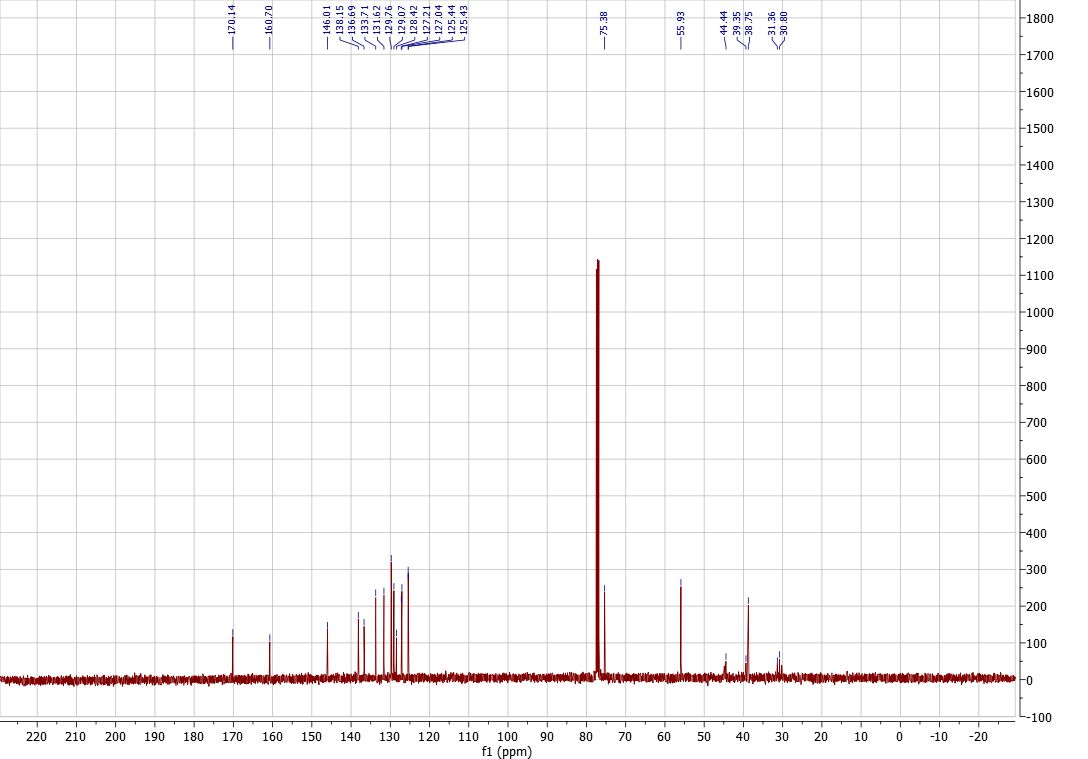
**

**4-(4-fluoro-3-(4-methoxypiperidine-1-carbonyl)benzyl)phthalazin-1(2H)-one (AZD2461)**

**
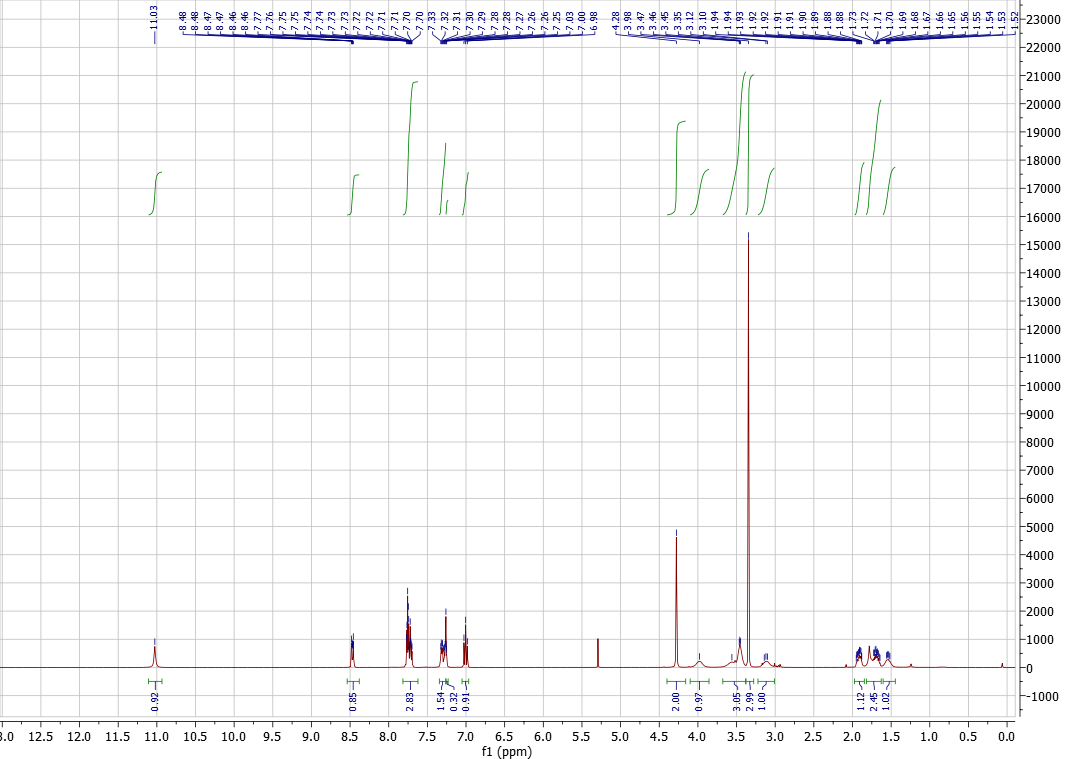
**

**
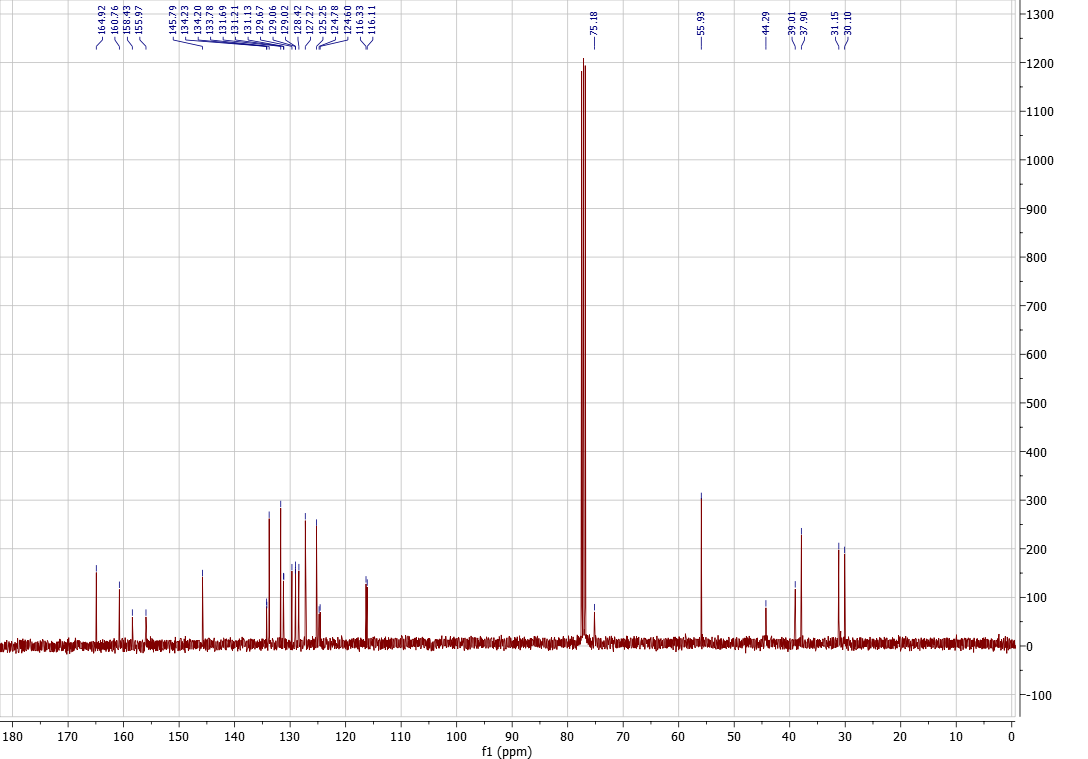
**

**
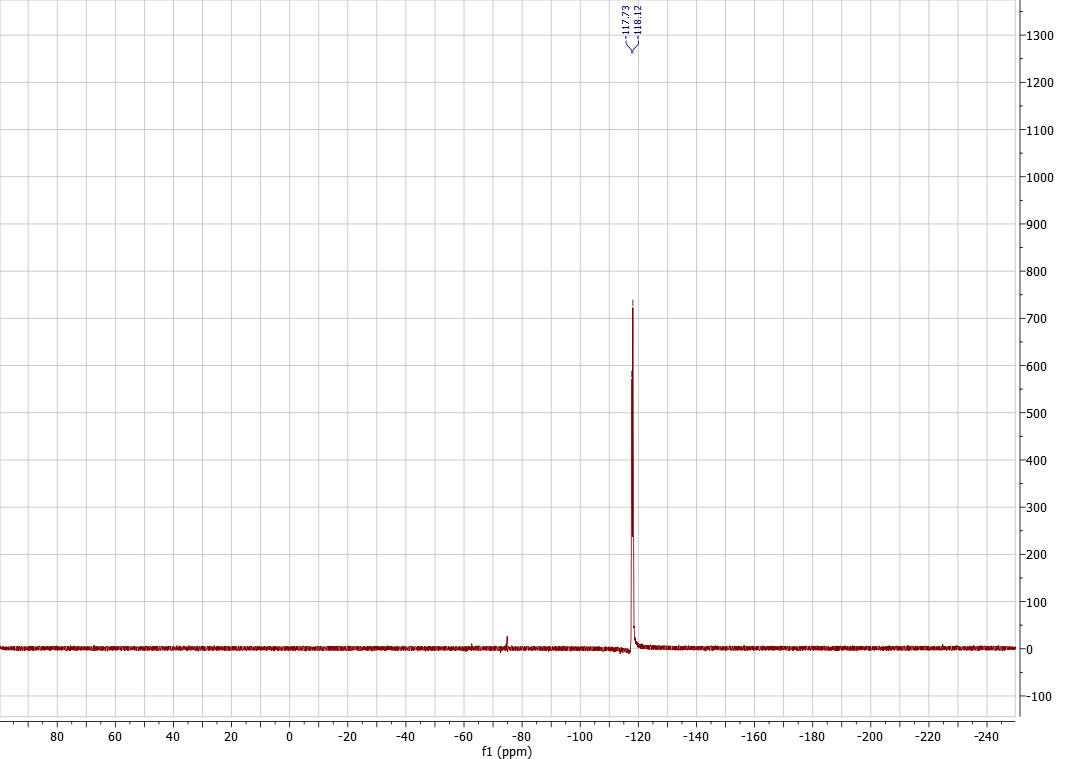
**

**REFERENCES for supplemental material**

1. Wilson TC, Xavier MA, Knight J, et al. (2019) PET Imaging of PARP Expression Using (18)F-Olaparib. J Nucl Med 60:504-510.

2. Guibbal F, Isenegger PG, Wilson TC, et al. (in press) Manual and Automated Cu-Mediated Radiosynthesis of the PARP Inhibitor [18F]Olaparib. Nature protocols.
